# Animal-assisted therapy for patients in a minimally conscious state: A randomized two treatment multi-period crossover trial

**DOI:** 10.1371/journal.pone.0222846

**Published:** 2019-10-01

**Authors:** Karin Hediger, Milena Petignat, Rahel Marti, Margret Hund-Georgiadis

**Affiliations:** 1 Department of Psychology, Division of Clinical Psychology and Psychotherapy, University of Basel, Basel, Switzerland; 2 REHAB Basel, Clinic for neurorehabilitation and paraplegiology, Basel, Switzerland; 3 Department of Epidemiology and Public Health, Human and Animal Health Unit, Swiss Tropical and Public Health Institute, Basel, Switzerland; 4 Institute for Interdisciplinary Research on the Human-Animal Relationship Switzerland, Basel, Switzerland; IRCCS E. Medea, ITALY

## Abstract

**Objective:**

To investigate if animal-assisted therapy (AAT) leads to higher consciousness in patients in a minimally conscious state during a therapy session, measured via behavioral reactions, heart rate and heart rate variability.

**Methods:**

In a randomized two treatment multi-period crossover trial, 10 patients in a minimally conscious state participated in eight AAT sessions and eight paralleled conventional therapy sessions, leading to 78 AAT and 73 analyzed control sessions. Patients’ responses during sessions were assessed via behavioral video coding and the Basler Vegetative State Assessment (BAVESTA), heart rate and heart rate variability (SDNN, RMSSD, HF and LF). Data were analyzed with generalized linear mixed models.

**Results:**

Patients showed more eye movements (IRR = 1.31, 95% CI: 1.23 to 1.40, *p* < 0.001) and active movements per tactile input during AAT compared to control sessions (IRR = 1.13, 95% CI: 1.02 to 1.25, *p* = 0.018). No difference was found for positive emotions. With BAVESTA, patients’ overall behavioral reactions were rated higher during AAT (b = 0.11, 95% CI: 0.01 to 0.22, *p* = 0.038). AAT led to significantly higher LF (b = 5.82, 95% CI: 0.55 to 11.08, *p* = 0.031) and lower HF (b = -5.80, 95% CI: -11.06 to -0.57, *p* = 0.030), while heart rate, SDNN, RMSSD did not differ.

**Conclusions:**

Patients in a minimally conscious state showed more behavioral reactions and increased physiological arousal during AAT compared to control sessions. This might indicate increased consciousness during therapeutic sessions in the presence of an animal.

**Trial registration:**

ClinicalTrials.gov NCT02629302.

## Introduction

Acquired brain injuries can result in severe disorders of consciousness, such as minimally conscious state (MCS), with often serious lifelong consequences for patients and their families [1–3]. Early onset of rehabilitation is a crucial factor with the goal of enhancing the patient’s consciousness by creating learning possibilities [4,5]. Current treatment concepts focus on stimuli which are activity-oriented and relevant for the individual patients, because personally and emotionally relevant stimuli induce higher-level activation in patients with disorders of consciousness [6,7]. Since animals are highly emotionally relevant [8], animal-assisted therapy (AAT) is an increasingly utilized approach in neurorehabilitation. AAT is a goal-directed intervention, in which a trained animal is an integral part of therapeutic activities [9]. Although there is anecdotal practical evidence [10], and AAT is becoming increasingly common in treatment of disorders of consciousness, empirical evidence from randomized controlled studies is lacking. Therefore, the aim of this study was to examine the effect of AAT on consciousness in patients in a minimally conscious state compared to conventional standard therapy in a randomized controlled trial. To investigate effects on patients’ consciousness, we assessed patients’ behavior and measured physiological arousal via heart rate and heart rate variability.

## Methods

### Participants

**Fig 1:**
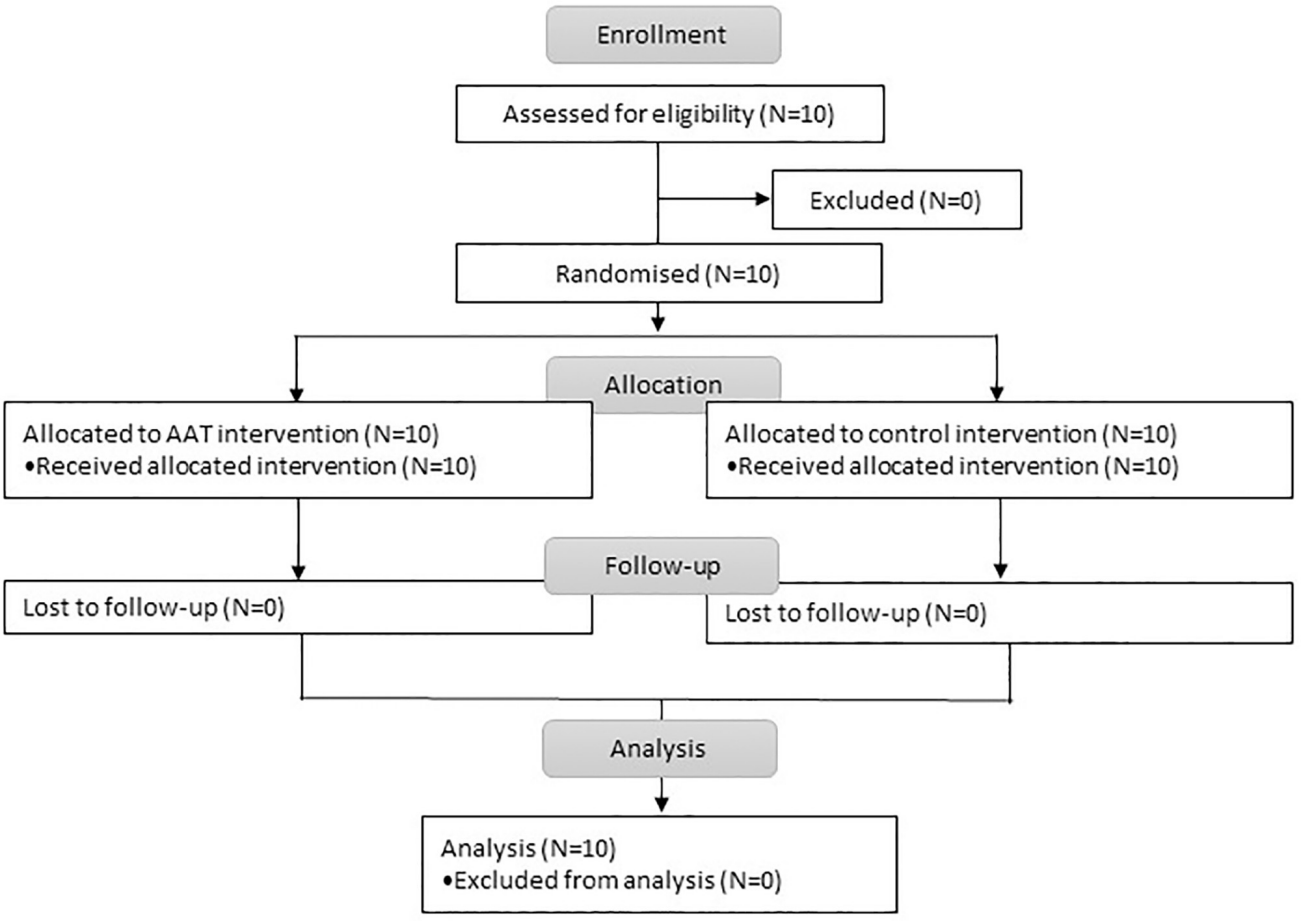
CONSORT flowchart.

Subjects were 10 inpatients in a minimally conscious state. All patients were in stationary neurorehabilitation in a Swiss rehabilitation clinic, aged 17 to 71 years (*M* = 47.20, *SD* = 19.36) and diagnosed with acquired brain injury with either traumatic (N = 4) or non-traumatic causes (N = 6). Secondary diagnoses were not considered. Severity of the disorder of consciousness was assessed via the original JFK Coma Recovery Scale (CRS) [11,12] that does not include all of the behavioral criteria necessary to diagnose the minimally conscious state. The diagnosis was therefore based on clinical assessment by the responsible physician according to the Aspen diagnostic criteria [13] and to Bruno and colleagues [14] for the division of MCS+ and MCS-. MCS+ is characterized by the presence of command following, intelligible verbalization or gestural or verbal yes/no responses. MCS- patients in contrast only show minimal levels of behavioral interaction characterized by the presence of non-reflex movements. Patients were eligible for participation in the study if the scores and clinical assessment indicated a minimally conscious state following an acquired brain injury. Exclusion criteria were medical contraindications, such as phobias and allergies, assessed via interviews with relatives. The data was collected from May 2015 until April 2017. Fig 1 shows the CONSORT flowchart.

### Standard protocol approvals, registrations, and participant consents

The screening process involved the family members of the patients as well as the responsible physicians and therapists. The legal representative of the patients provided written informed consent. The human-related protocols were approved by the Ethics Committee for Northwest and Central Switzerland and the animal-related protocols were approved by the Veterinary Office of the Canton Basel-Stadt, Switzerland. AAT was performed according to the guidelines of the International Association of Human Animal Interaction Organizations (IAHAIO) to ensure patient safety and animal welfare [9]. No therapy session had to be ended early and no adverse incidents occurred. After participating in the study, all patients had the possibility to continue with AAT. The study was registered at ClinicalTrials.gov (Identifier: NCT02629302).

### Study design and procedure

The study was designed as a randomized two treatment multi-period crossover design to evaluate the immediate effects of the different interventions on patient reactions. Standardized therapy sessions that integrated an animal served as experimental condition and are referred to as AAT sessions. In the control condition, paralleled, comparable standardized therapy sessions without the presence of an animal (treatment as usual) were used. Each patient participated in 16 therapy sessions over a period of 4 weeks (*N* AAT = 8, *N* control = 8). Sessions lasted for approximately 15 minutes and were held four times a week, twice with an animal and twice without an animal. Each control session was paralleled with an AAT session such that two sessions in two consecutive weeks were as similar as possible regarding the involved therapist, day of the week, time of day and therapeutic activity. All participants were allocated randomly to start with either AAT or a control session. Allocation sequence was generated via a random number generator by the principal investigator who also enrolled and assigned participants to interventions. Some of the originally planned 160 sessions were cancelled due to illness of patient or therapist, and for some sessions data was lost due to technical problems. In total, we coded the behavior of 151 sessions (*N* AAT = 78, *N* control = 73) and analyzed assessment data of 136 sessions (*N* AAT = 69, *N* control = 67) and heart rate data of 115 sessions (*N* AAT = 61, *N* control = 54). All AAT and control sessions were held in a therapy room within the therapy animal facility at the rehabilitation center. The patients were transported to the therapy room by wheelchair. Patients wore a heart rate monitor belt on their chest which continuously measured heart rate and heart rate variability during the session. All sessions were videotaped and at the end of each session, the behavior of the patients was assessed via the Basler Vegetative State Assessment by the therapists. Prior to the study start, a suitable animal was selected for each patient according to preference and abilities. Included species were dogs, guinea pigs and rabbits. All animals were trained for AAT, had experience working with patients in a minimally conscious state, and were kept and handled according to the IAHAIO standards [9]. Guinea pigs and rabbits were put into a table cage where they could interact with patients or retreat at will. During the AAT sessions, therapeutic activities were performed by physically guiding the patient’s hands according to the Affolter concept [15]. Examples of therapeutic activities were: brushing a dog, cutting vegetables and feeding them to the rabbits or guinea pigs, or opening a box with herbs and feeding them to the rabbits or guinea pigs. Paralleled control sessions consisted of therapeutic interventions with basic activities selected from a range of occupational therapy assignments. These activities were also performed according to the Affolter concept. Corresponding examples of control activities were: brushing a fake fur, preparing food by cutting vegetables and putting them in a bowl, or opening an empty box and filling it.

### Behavioral analysis via video coding

As primary outcome, the patient’s reactions was assessed via behavioral video coding. Therapy sessions (*N* = 151) were videotaped with a handheld camera (Sony HDR-CX240) and analyzed with a behavioral coding system software (Observer XT 12, Noldus). Analyses were done continuously, defining each second of the video with the different variables as present or not for state behavior variables. We calculated the percentage of the duration of each state variable in relation to the observed time period of a therapy session. Count variables were coded only if they occurred, and the total occurrence within a therapy session was calculated. All videos were coded according to a strict ethogram defined by detailed descriptions of the behaviors with inclusion and exclusion examples. The coding scheme was developed for the purpose of this study. As basis, 11 existing paper-pencil behavioral assessment tools in German and English for patients with disorders of consciousness were screened. Items were pooled and reduced to behaviors that could be observed during video analysis and that occur according to a stimulus during a therapeutic situation. Our coding scheme included the dimensions “eyes open/closed”, “eye movement”, “movement”, “phonation” and “emotion” (operationalized via facial expression). Moreover, we coded the amount of verbal and tactile stimuli offered by the therapist as well as the amount of the patient’s physical contact with the animal. Inter-rater reliability was measured by Cohen’s kappa for all coded variables. Before coding the actual data, each rater achieved an inter-rater reliability of *k* > 0.80. Inter-rater reliability ranged between 0.83 and 0.99 indicating excellent agreement among coders.

### Basler Vegetative State Assessment

The Basler Vegetative State Assessment (BAVESTA) [16], a behavioral assessment tool for patients with disorders of consciousness, was used as an additional tool to measure behavioral reactions of patients during each therapy session and served as secondary outcome. This study used 22 of the original 33 items, targeting behaviors that are observable during a short period of time, and adjusted the calculations of total short-term mean score and short-term subscores accordingly, with a range from 0 (behavior is not shown) to 5 (behavior is consistently shown). After each therapy session, the therapist assessed the patient with this short-term BAVESTA.

### Heart rate and heart rate variability recording

Heart rate (HR) and heart rate variability (HRV) were measured using non-invasive HR monitoring belts (Polar^®^ RS800CX, Polar^®^ Electro Oy) as further secondary outcomes. The recorded inter-beat intervals were analyzed with Kubios HRV analysis software version 3.0.2 (Biosignal Analysis and Medical Imaging Group, University of Kuopio, Finland). In each therapy session, a 5-minute recording was selected. In control sessions, the 5-minute sequence was taken from the middle of the whole session. For AAT sessions, the duration of interaction between the patient and the animal was identified via the videos and the 5-minute sequence was taken from the middle of the interaction phase. Before processing, all RR-series were visually checked and, when necessary, artifacts were corrected. If the number of corrected beats was higher than 5%, the data was excluded from analysis (*N* AAT = 1, *N* control = 2). We also excluded data if the total recording or the interaction between the patient and the animal was shorter than 5 minutes (*N* = 1). We calculated the following HRV parameters: time domain: the standard deviation of all normal-to-normal RR intervals (SDNN, ms) and root-mean square differences of successive RR intervals (RMSSD, ms); and frequency domain: relative power of the low frequency (LF) and high frequency (HF) band in normal units.

### Statistical analysis

Behavior analysis was performed using generalized linear mixed models. Count data were modeled as rates using a Poisson distribution and the logarithm of the duration of the sessions as an offset variable. The primary models included only the outcome variable and the treatment type as single predictor. Participant IDs were included as random effect to account for multiple observations within each subject. The Incident Rate Ratio (IRR) was used as effect size. The model holds under the assumption that there is no time effect which might be violated. Therefore, we checked the robustness of the model by fitting a second model equivalent to the previous one but including session number as a categorical fixed effect. During data inspection we noticed that the therapists behave differently in AAT and control sessions, primarily with respect to the number of tactile inputs. Because those inputs trigger most of the patients’ reactions, we fitted a third model that includes time as well as the log of tactile inputs. For descriptive statistics, the number of observed count behaviors (count variables) was transformed into rate per time ((n/time)*100 sec) and rate per tactile inputs ((n/tactile inputs)*100). To analyze the effect of AAT on BAVESTA scores and HR/HRV parameters as secondary outcomes, generalized linear mixed models with condition as fixed effect and the individual patient as random effect with the mean difference (b) as effect size was used. All variables were visually checked to detect extreme values (histogram and Q-Q-plot). Model diagnostics of linear mixed models included visual checks for normality of residuals and homogeneity of residuals. All residuals were approximately normally distributed with the exception of RMSSD, which was therefore log-normal transformed. No data were excluded except for HR/HRV data with corrected beats greater than 5% and recordings where patient and animal interacted for less than 5 minutes. Sample size was estimated based on clinical experience and on a pre-analysis of an ongoing study. The significance level was set at the 5% level and all statistical analyses were performed using SPSS, Version 24, and R, Version 3.5.1.

## Results

Two female and eight male participants, between age 17 to 71 with an average age of 47 years (*M* = 47.20, *SD* = 19.36), participated in this study. CRS values at study start ranged between 14 and 22. All patients were diagnosed with MCS in a clinical assessment by the responsible physician according to the Aspen diagnostic criteria [13] and the criteria of Bruno and colleagues [14]. Table 1 summarizes the principal clinical and demographic characteristics of participants. Table 2, S1 and S2 Tables provide an overview of the intervention characteristics.

**Table 1:** Sample characteristics.

Subject	Gender	Age	Etiology	Main pathology	Days since event	Admission	CRS^✝^	Diagnosis*
1	Male	71	TBI	Polytrauma	265	Initial rehabilitation	22	MCS+
2	Female	60	nonTBI	Subarachnoid hemorrhage	114	Initial rehabilitation	22	MCS+
3	Male	61	nonTBI	Cerebrovascular ischemia	103	Initial rehabilitation	21	MCS+
4	Female	27	nonTBI	Cerebrovascular ischemia	102	Initial rehabilitation	15	MCS-
5	Male	27	TBI	Polytrauma	2654	Readmission	17	MCS-
6	Male	17	TBI	Polytrauma	120	Initial rehabilitation	17	MCS-
7	Male	70	TBI	Subarachnoid hemorrhage	83	Initial rehabilitation	17	MCS+
8	Male	57	nonTBI	Subarachnoid hemorrhage	138	Initial rehabilitation	16	MCS+
9	Male	37	nonTBI	Hypoxic and metabolic encephalopathy	105	Initial rehabilitation	14	MCS-
10	Male	45	nonTBI	Hypoxic-ischemic encephalopathy	4979	Readmission	17	MCS-

TBI: traumatic brain injury, CRS: JFK Coma Recovery Scale total score at study start, MCS: minimally conscious state,

^✝^refers to the original, not the revised instrument with a maximum total score of 25,

*diagnosis according to the Aspen Workgroup criteria and the criteria of Bruno et al., 2011.

**Table 2:** Intervention characteristics.

**Variable**	****	**AAT**	**Control**	**AAT (%)**	**Control (%)**
Therapy time	Morning	28	25	52.83	47.17
	Afternoon	50	47	51.55	48.45
**Variable**		**AAT M**	**Control M**	**AAT SD**	**Control SD**
Video length*		887.50	855.79	199.51	189.24
Total number of tactile input		114.19	148.04	57.35	71.51
Total amount of verbal input*		251.77	287.00	184.36	209.35

AAT: animal-assisted therapy, M: mean, SD: standard deviation,

* in seconds

### Behavior analysis

There were more tactile inputs from therapists during control sessions than during AAT sessions (control: M = 148.04, SD = 71.51, AAT: M = 114.19, SD = 57.35; IRR = 0.74, 95% CI: 0.68 to 0.81, *p* < 0.001), while verbal inputs from therapists did not differ significantly between conditions (control: M = 33.86, SD = 21.45, AAT: M = 28.66, SD = 18.86; b = -0.05, 95% CI: -0.11 to 0.01, *p* = 0.074).

Patients showed a significantly higher rate of eye movement of 5 movements per 100 seconds during AAT compared to control therapy sessions with a rate of 4 (IRR = 1.17, 95% CI: 1.11 to 1.24, *p* < 0.001). This effect was also present for the models that include time or time and tactile input. The rate of eye movement per tactile input even increased by a factor of 1.7 during AAT compared to control therapy sessions. The rate of total movements per 100 seconds was higher during control therapy sessions and decreased from 3.5 to 3 during AAT (IRR = 0.85, 95% CI: 0.80 to 0.91, *p* < 0.001). However, this effect reversed when time and tactile inputs were added to the model (IRR = 1.08, 95% CI: 1.00 to 1.16, *p* = 0.048) with a rate of 23 during AAT and a rate of 20 movements per 100 seconds during control therapy sessions. While there was no difference in self-initiated (active) movements in model one or two, patients showed significantly more self-initiated movements per tactile input during AAT compared to control therapy sessions (IRR = 1.13, 95% CI: 1.02 to 1.25, *p* = 0.018). The opposite effect was found for reactive movements of the patients. The rate of reactive movements per time was lower during AAT sessions (IRR = 0.74, 95% CI:0.67 to 0.80, *p* < 0.001) but this difference disappeared when looking at the rate per tactile input. Patients showed a higher amount of phonation during AAT compared to control therapy sessions (IRR = 1.92, 95% CI: 1.32 to 2.78, *p* < 0.001) but again, this effect disappeared when time and tactile inputs were included in the model. There was no difference regarding positive emotions, operationalized via positive facial expressions. Negative emotions were reduced during AAT compared to control therapy sessions but this difference was only statistically significant when the amount of tactile inputs were taken into account (IRR = 0.35, 95% CI: 0.20 to 0.59, *p* < 0.001, see Table 3).

**Table 3:** Analyzed behaviors during AAT and control therapy sessions.

							Model 1			Model 2			Model 3		
Behavior	Setting	N	M	SD	Rate time	Rate input	IRR	95% CI	p-value	IRR	95% CI	p-value	IRR	95% CI	p-value
Eye movement^+^	Control	73	26.71	29.68	3.99	18.04	1.17	1.11 to 1.24	<0.001*	1.17	1.10 to 1.25	<0.001*	1.31	1.23 to 1.40	<0.001*
	AAT	78	35.47	37.46	5.05	31.07									
Movement total	Control	73	30.03	31.79	3.51	20.28	0.85	0.80 to 0.91	<0.001*	0.87	0.82 to 0.93	<0.001*	1.08	1.00 to 1.16	0.048*
	AAT	78	26.06	29.17	2.94	22.82									
Movement active	Control	73	14.95	14.68	1.75	10.10	0.97	0.89 to 1.05	0.441	0.95	0.86 to 1.04	0.240	1.13	1.02 to 1.25	0.018*
	AAT	78	14.97	17.55	1.69	13.11									
Movement reactive	Control	73	15.08	24.68	1.76	10.19	0.74	0.67 to 0.80	<0.001*	0.78	0.71 to 0.87	<0.001*	0.98	0.88 to 1.10	0.756
	AAT	78	11.09	17.65	1.25	0.71									
Phonation	Control	73	0.60	1.61	0.07	0.41	1.92	1.32 to 2.78	<0.001*	1.38	0.87 to 2.18	0.173	1.23	0.74 to 2.07	0.423
	AAT	78	0.96	4.51	0.11	0.84									
Positive facial expression	Control	73	1.19	2.65	0.14	0.81	1.14	0.85 to 1.52	0.382	1.10	0.79 to 1.54	0.567	1.05	0.72 to 1.55	0.795
	AAT	78	1.23	32.48	0.14	1.09									
Negative facial expression	Control	73	2.10	6.57	0.24	1.42	0.86	0.68 to 1.08	0.200	0.71	0.48 to 1.06	0.096	0.35	0.20 to 0.59	<0.001*
	AAT	78	1.78	6.11	0.20	1.54									

AAT: animal-assisted therapy, N: number of analyzed sessions, M: mean (absolute), SD: standard deviation, rate time: rate per 100 seconds, rate input: rate per 100 tactile inputs, IRR: Incident Rate Ratio, CI: confidence interval, Model 1: therapy type as fixed effect, Model 2: therapy type and time as fixed effect, Model 3: therapy type, time and log tactile input as fixed effect,

*statistically significant,

^+^ log of the time when eyes were observable was used as offset to analyze eye movement.

### Basler Vegetative State Assessment

In the BAVESTA, the patients overall behavioral reactions were rated higher during AAT sessions compared to control sessions (b = 0.11, 95% CI: 0.01 to 0.22, *p* = 0.038). While there was no difference regarding the subscales “attention”, “verbal communication” “emotional reactions” or “motor reactions”, we found significantly higher perception and information processing scores (perception: b = 0.21, 95% CI: 0.01 to 0.41, *p* = 0.041; information processing: b = 0.19, 95% CI: 0.03 to 0.34, *p* = 0.023) as well as significantly more nonverbal communication (b = 0.19, 95% CI: 0.05 to 0.33, *p* = 0.010) during AAT compared to standard therapy sessions (see Table 4).

**Table 4:** Basler Vegetative State Assessment after AAT and control therapy sessions.

Scale	Setting	N	M	SD	b	95% CI	p-value
BAVESTA total	Control	67	1.91	0.30	0.11	0.01 to 0.22	0.038*
	AAT	69	2.03	0.49			
Attention	Control	67	3.28	0.64	0.12	-0.10 to 0.34	0.289
	AAT	69	3.39	0.84			
Perception	Control	67	2.60	0.62	0.21	0.01 to 0.41	0.041*
	AAT	69	2.82	0.83			
Emotional reactions	Control	67	1.75	0.93	0.27	-0.01 to 0.56	0.061
	AAT	69	2.01	1.18			
Nonverbal communication	Control	67	1.78	0.44	0.19	0.05 to 0.33	0.010*
	AAT	69	1.97	0.66			
Verbal communication	Control	67	0.63	0.27	-0.04	-0.13 to 0.04	0.321
	AAT	69	0.60	0.28			
Motor reactions	Control	67	1.09	0.36	0.07	-0.04 to 0.19	0.219
	AAT	71	1.15	0.46			
Information processing	Control	67	1.90	0.52	0.19	0.03 to 0.34	0.023*
	AAT	69	2.08	0.68			

Scales are adapted and only include items targeting short-term behavior. AAT: animal-assisted therapy session, N: number of analyzed sessions, M: mean, SD: standard deviation, b: mean difference, CI: confidence interval,

*statistically significant

### Heart rate / heart rate variability

Heart rate as well as heart rate variability parameters SDNN and RMSSD did not differ significantly between AAT and control sessions (see Table 5). In contrast, patients showed significantly higher LF (b = 5.82, 95% CI: 0.55 to 11.08, *p* = 0.031) and lower HF values (b = -5.80, 95% CI: -11.06 to -0.57, *p* = 0.030) during AAT compared to control sessions.

**Table 5:** Heart rate and heart rate variability.

Parameter	Setting	N	M	SD	b	95% CI	p-value
HR, bpm	Control	54	80.22	17.22	0.898	-1.143 to 3.23	0.446
	AAT	61	80.81	16.75			
SDNN, ms	Control	54	22.82	18.79	-1.37	-5.41 to 2.67	0.503
	AAT	61	20.34	15.66			
RMSSD, ms^+^	Control	54	22.20	29.12	-0.06	-0.26 to 0.15	0.601
	AAT	61	17.90	22.67			
LFnu	Control	54	64.77	27.22	5.82	0.55 to 11.08	0.031*
	AAT	61	68.87	24.00			
HFnu	Control	54	35.12	27.16	-5.80	-11.06 to -0.57	0.030*
	AAT	61	31.04	23.94			

HR: mean heart rate; bpm: beats per minute; SDNN: the standard deviation of all normal-to-normal RR intervals; RMSSD: root-mean square differences of successive RR intervals; pNN50: percentage of successive normal RR intervals exceeding 50 ms; LF: low frequency; HF: high frequency; nu: normalized units; PA: physical activity, AAT: animal-assisted therapy session, N: number of analyzed sessions, M: mean, SD: standard deviation, b: coefficient, CI: confidence interval,

^+^absolute data is presented, while the model was run with ln transformed data;

*statistically significant

## Discussion

We present the first randomized controlled trial of patients in a minimally conscious state assessing behavioral reactions and arousal during AAT and control therapy sessions. AAT led to significantly more eye movements, self-initiated movements as well as movements in total compared to control therapy sessions in the systematic behavior analysis. This is in line with results of Bardl and Bardl’s case-study [10] that documented improvements in visual exploration, spontaneous reactions and target-oriented movements in a patient in a persistent vegetative state during the presence of a dog, as well as Jones, Rice and Cottons’ review who showed increased engagement during therapy due to AAT in adolescents with mental health disorders [17]. We did not find differences in positive emotional reactions which somewhat contrasts to previously published results. In the BAVESTA, patients had a higher total score during AAT indicating higher consciousness, and they showed more nonverbal communication and higher perception and information processing scores. No verbal communication was shown by most of the patients, so it is not surprising that we found no difference. The BAVESTA subscales “attention”, “emotional reactions” and “motor reactions” did not differ between the conditions. The observed effects of higher behavior reactions in the presence of an animal, measured using two different approaches, indicate a higher level of awareness [18], one of the two components of consciousness [19]. While the previous study involved a dog [10], our study documents that guinea-pigs and rabbits might have same beneficial effects as dogs.

We observed differences in the frequency domain heart rate variability parameters. During AAT sessions, HF values were significantly lower and values of LF were significantly higher compared to conventional standard therapy. The decrease in HF values reflects decreased activity of the parasympathetic nervous system, while the increase in LF values is associated with increased activity of the sympathetic nervous system [20,21], so both outcomes indicate higher physiological activity and an increase in arousal in the presence of an animal. Along with awareness, arousal is the other component of consciousness [19]. Increased arousal could therefore reflect a higher level of consciousness and indicate an underlying process that might explain the observed behavioral effects of the patients in the presence of an animal. Lowered values of HF have also been associated with mental activity and mental stress [22,23]. But the observed HF in the AAT condition was within the range of normal values [20] and the reduction might also indicate an increase in arousal associated with positive emotions, excitement and emotional involvement [24,25] rather than distress. However, since patients in a minimally conscious state are highly vulnerable, further research is needed to clarify these effects. Our findings are in line with a previous investigation documenting lower values of HF in autistic children following interaction with a live dog compared to a robotic dog [26]. However, there are mixed outcomes from studies, documenting no effects [27] or even higher heart rate variability as a result of an interaction with an animal [28]. We found no statistically significant difference in heart rate between AAT and treatment as usual. This is in contrast to studies documenting decreases in heart rate during animal-assisted interventions for a broad range of populations [29] or an increased heart rate in hospitalized children with chronic disorders prior to and following dog assisted therapy as compared to control therapy sessions [30].

Neither participants nor raters responsible for video coding could be blinded to the conditions. The crossover design of this study only allowed for detecting short-term effects of AAT on behavioral and heart rate measurements during therapy sessions, and the small sample size limits the study outcomes and warrants further trials with more patients. Strengths of this study are inclusion of a paralleled control condition, behavior measured with different approaches and inclusion of a physiological parameter to identify underlying mechanisms. Moreover, our results showed that the presence of an animal can also influence the behavior of the involved therapists and that patients’ reactions should be interpreted in relation to the behavior of the therapists. This is a relevant aspect that should be taken into account in further study designs.

### Conclusion

Our results indicate that AAT is a feasible approach to increase behavioral reactions and arousal in patients in a minimally conscious state. Integration of animals could be used to increase consciousness of these patients and lead to achieving a relevant therapeutic goal. Although this result is promising, the data are preliminary and it is necessary to further investigate whether AAT might be an effective approach to improve therapeutic effects of neurorehabilitation for patients in a minimally conscious state.

## Supporting information

S1 Fig Rate of eye movement during AAT and control therapy sessions over the course of the time. (TIF)

S2 Fig Rate of eye movement for each patient over the course of time. (TIF)

S3 Fig Rate of eye movement in the presence of different animals. (TIF)

S4 Fig Rate of total movement during AAT and control therapy sessions over the course of the time. (TIF)

S5 Fig Rate of total movement for each patient over the course of time. (TIF)

S6 Fig Rate of active movement during AAT and control therapy sessions over the course of the time. (TIF)

S7 Fig Rate of active movement for each patient over the course of time. (TIF)

S8 Fig Correlation matrix of the analyzed behaviors. (TIF)

S1 Table Sessions held by the different therapists. (DOCX)

S2 Table Allocation of therapy sessions to different days of the week. (DOCX)

S3 Table Correlations of the analyzed behaviors. (DOCX)

S1 File CONSORT checklist. (DOC)

S2 File Trial protocol. (PDF)
