# Supplementary figures and images for: Animal-assisted therapy for patients in a minimally conscious state: A randomized two treatment multi-period crossover trial
Source: PLoS One. 2019 Oct 1;14(10):e0222846. doi: 10.1371/journal.pone.0222846 (PMC6772068; doi:10.1371/journal.pone.0222846)

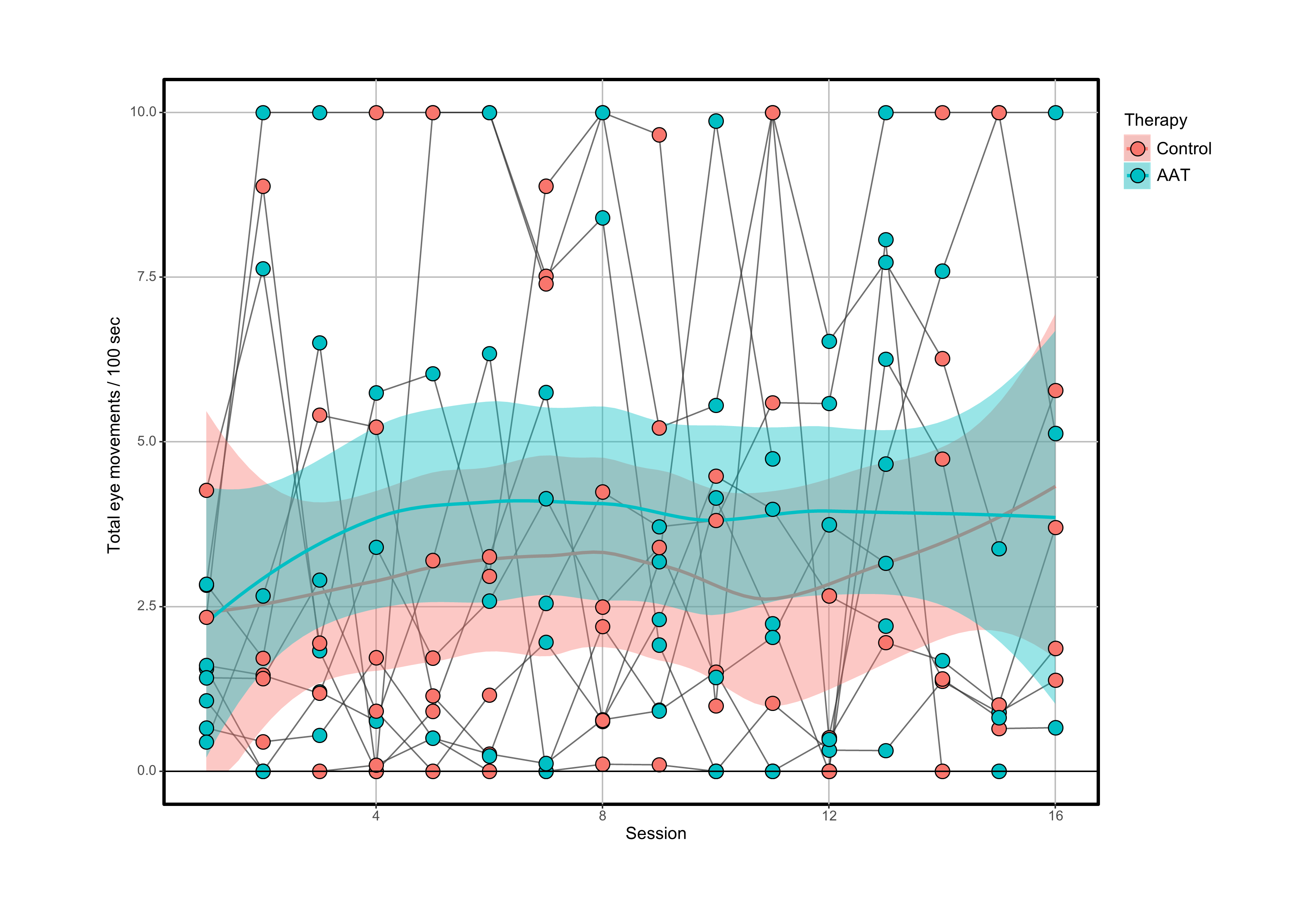

Supplement: S1 Fig — (TIF) [file pone.0222846.s001.tif]

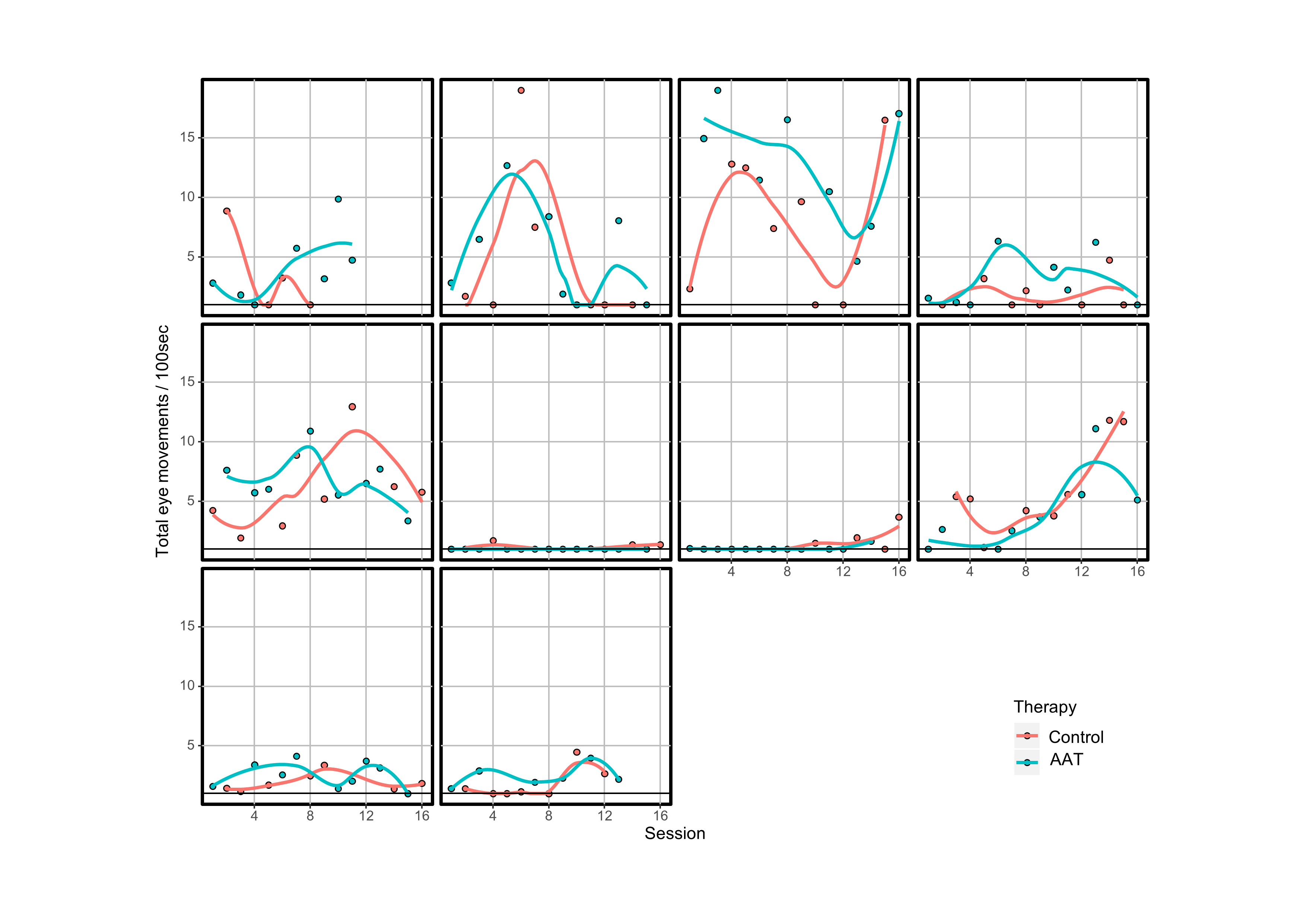

Supplement: S2 Fig — (TIF) [file pone.0222846.s002.tif]

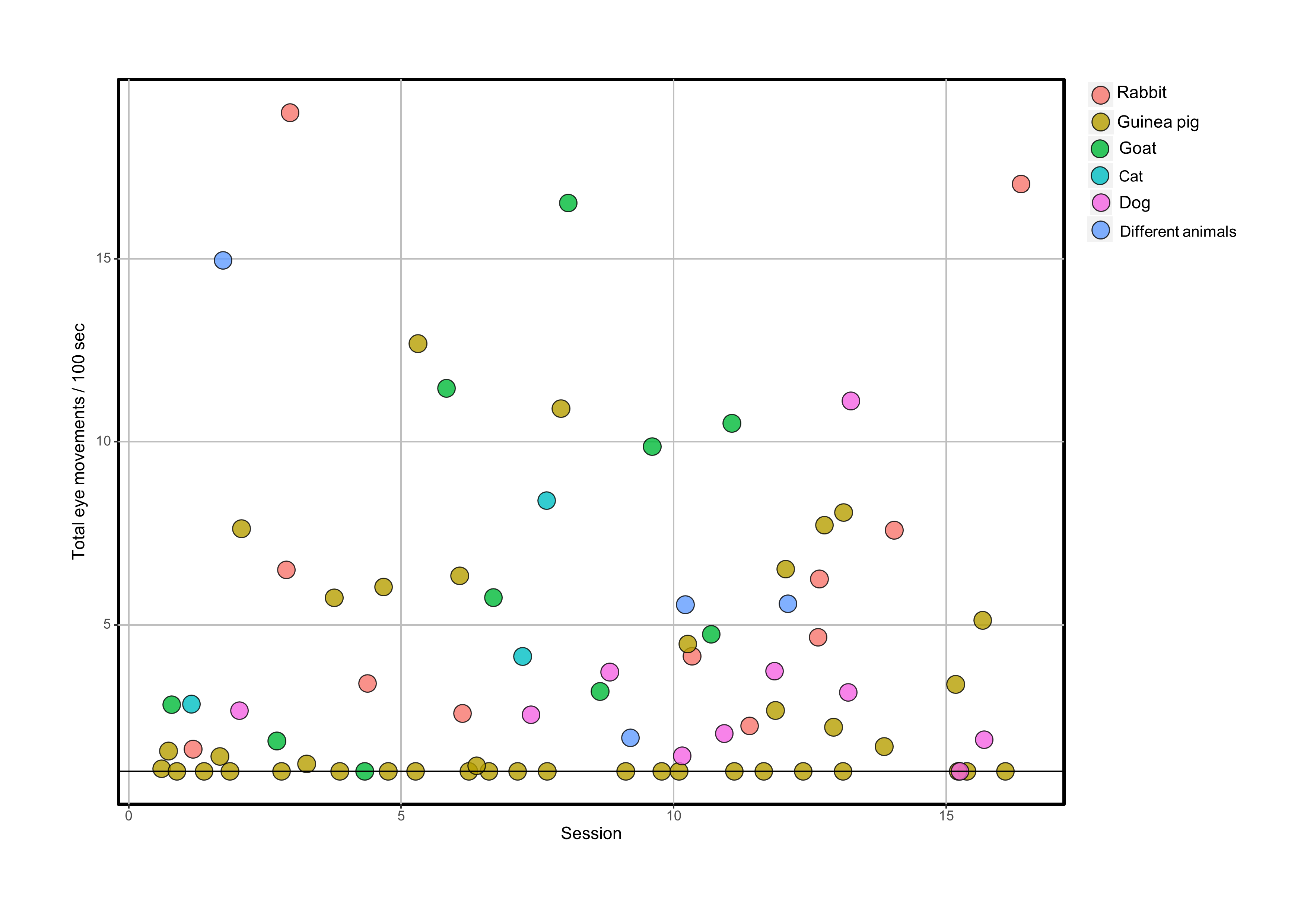

Supplement: S3 Fig — (TIF) [file pone.0222846.s003.tif]

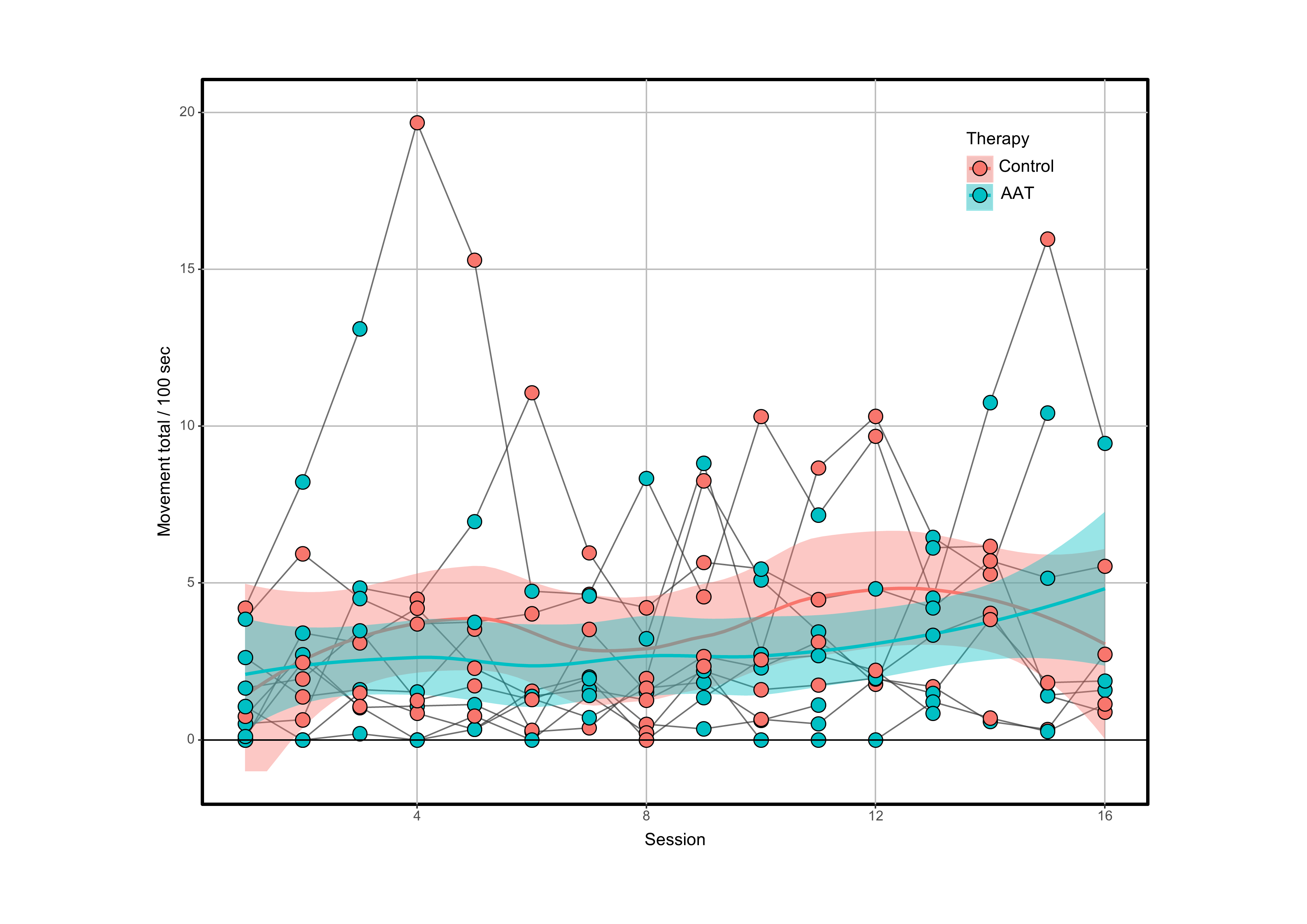

Supplement: S4 Fig — (TIF) [file pone.0222846.s004.tif]

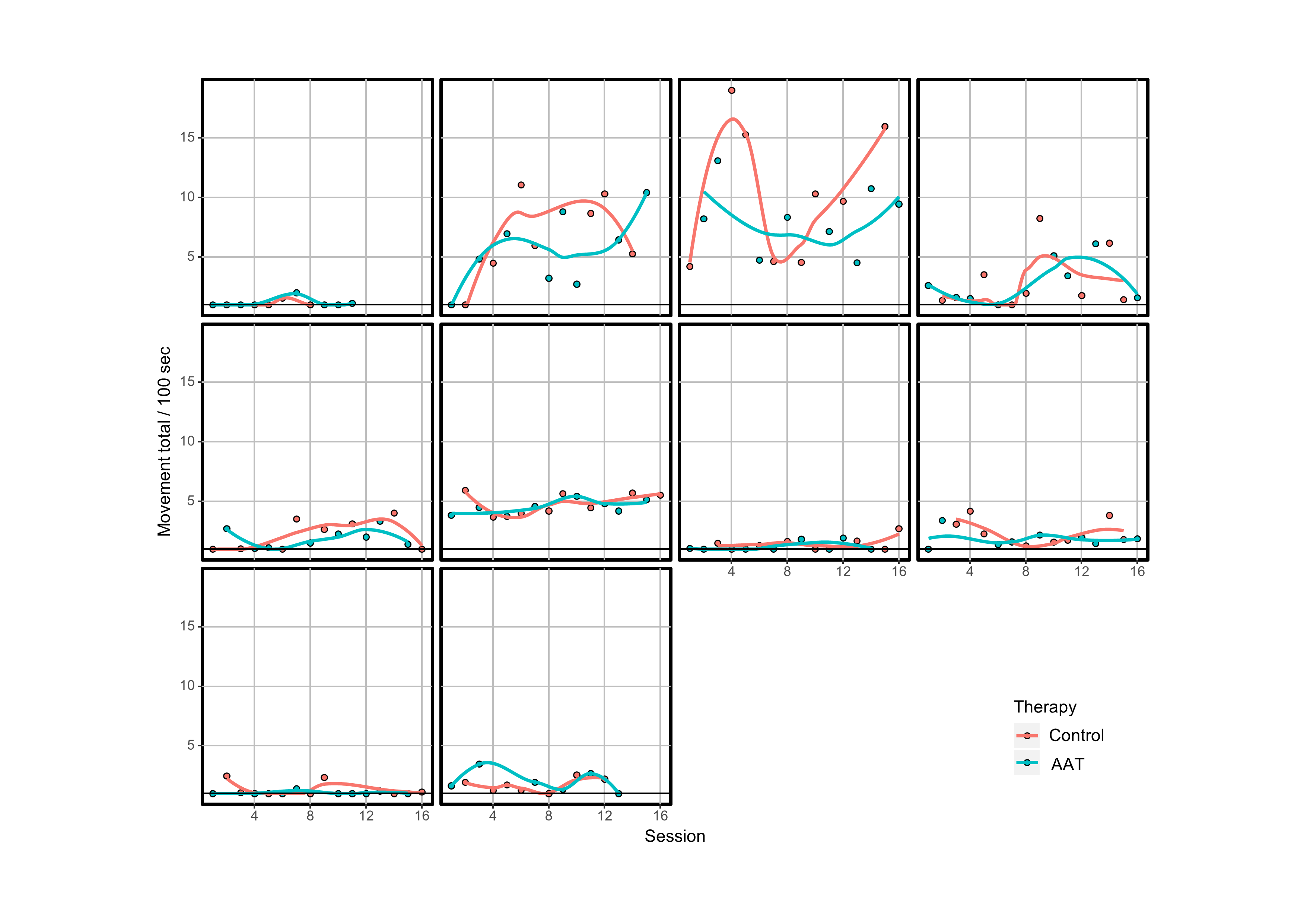

Supplement: S5 Fig — (TIF) [file pone.0222846.s005.tif]

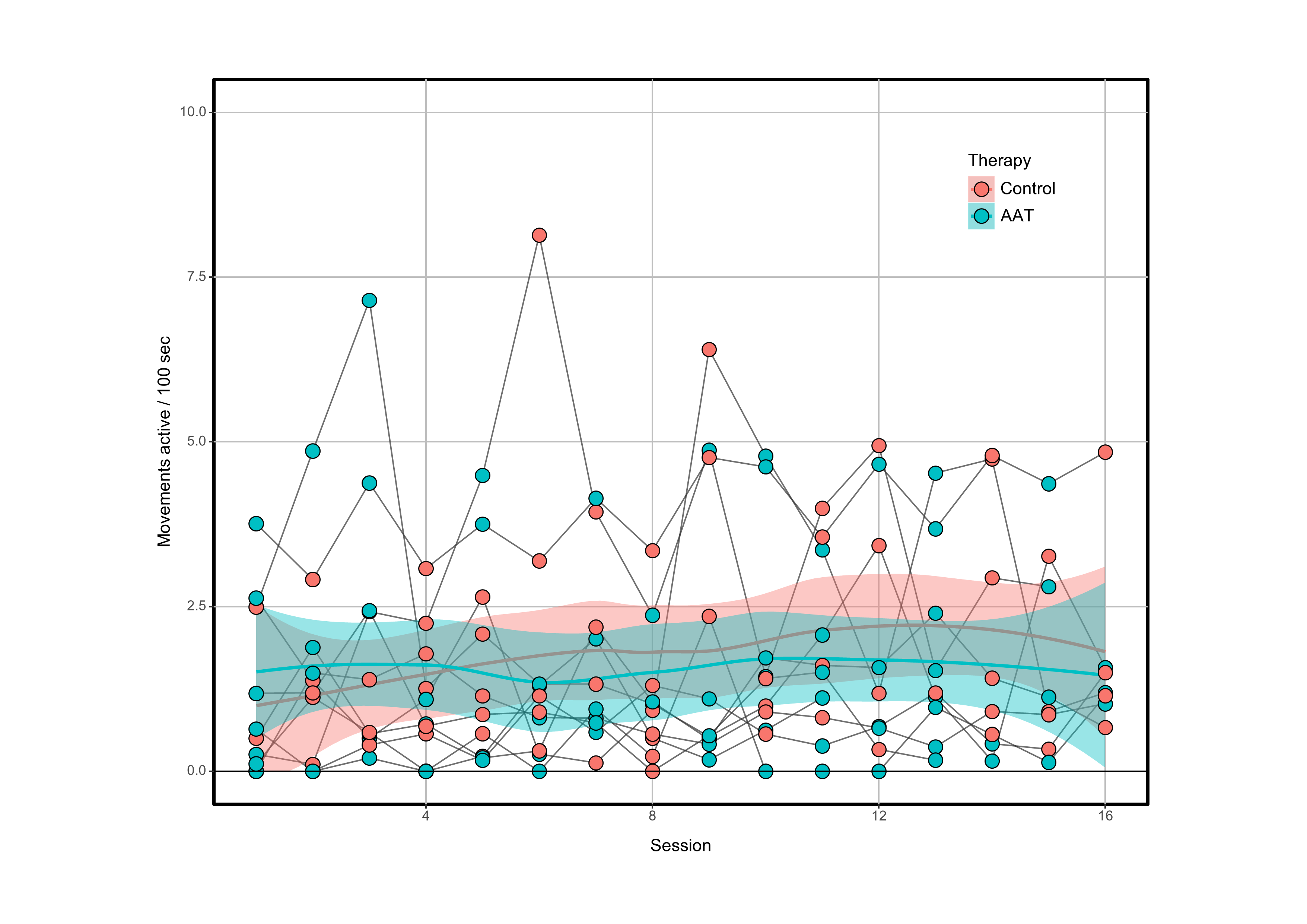

Supplement: S6 Fig — (TIF) [file pone.0222846.s006.tif]

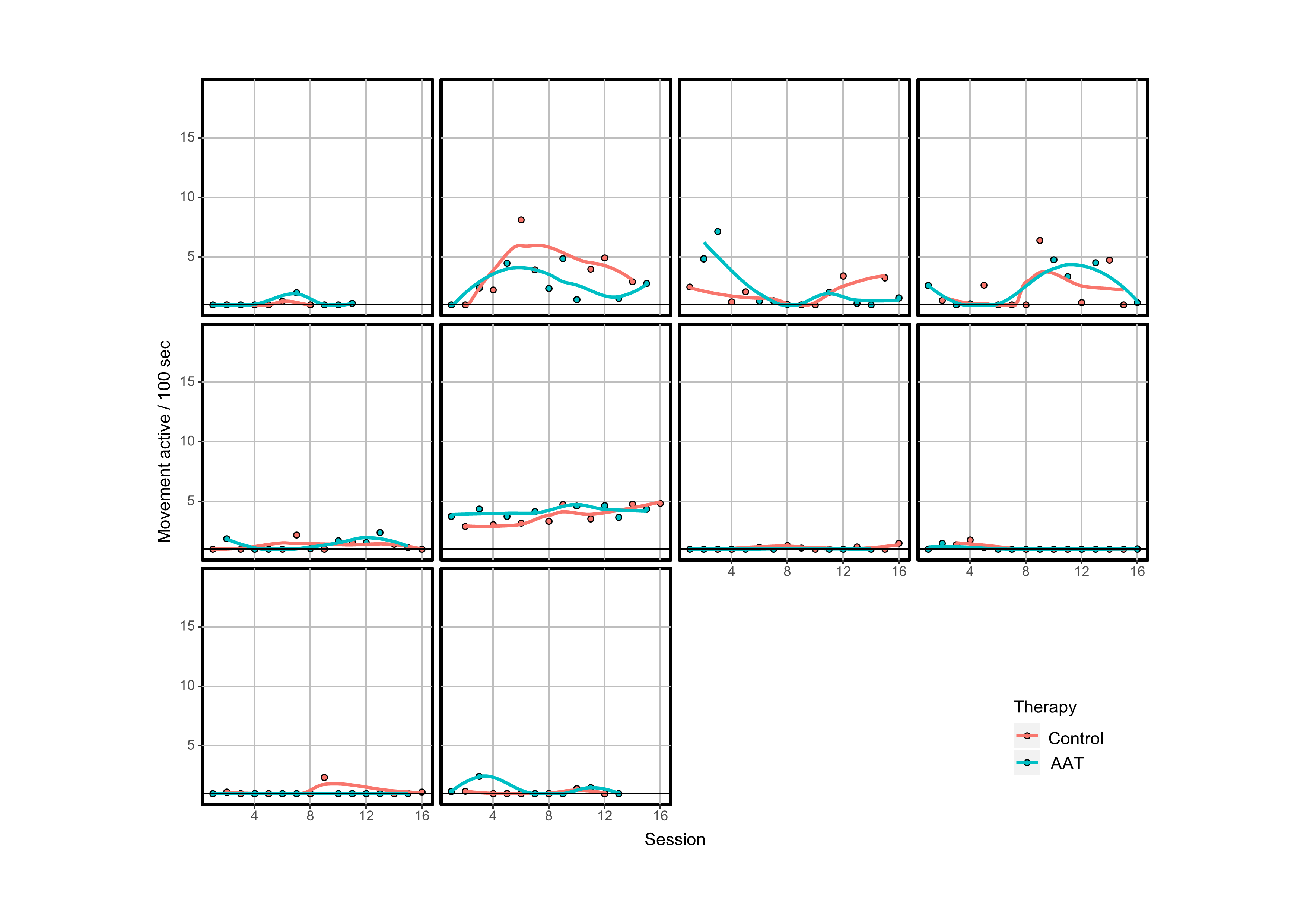

Supplement: S7 Fig — (TIF) [file pone.0222846.s007.tif]

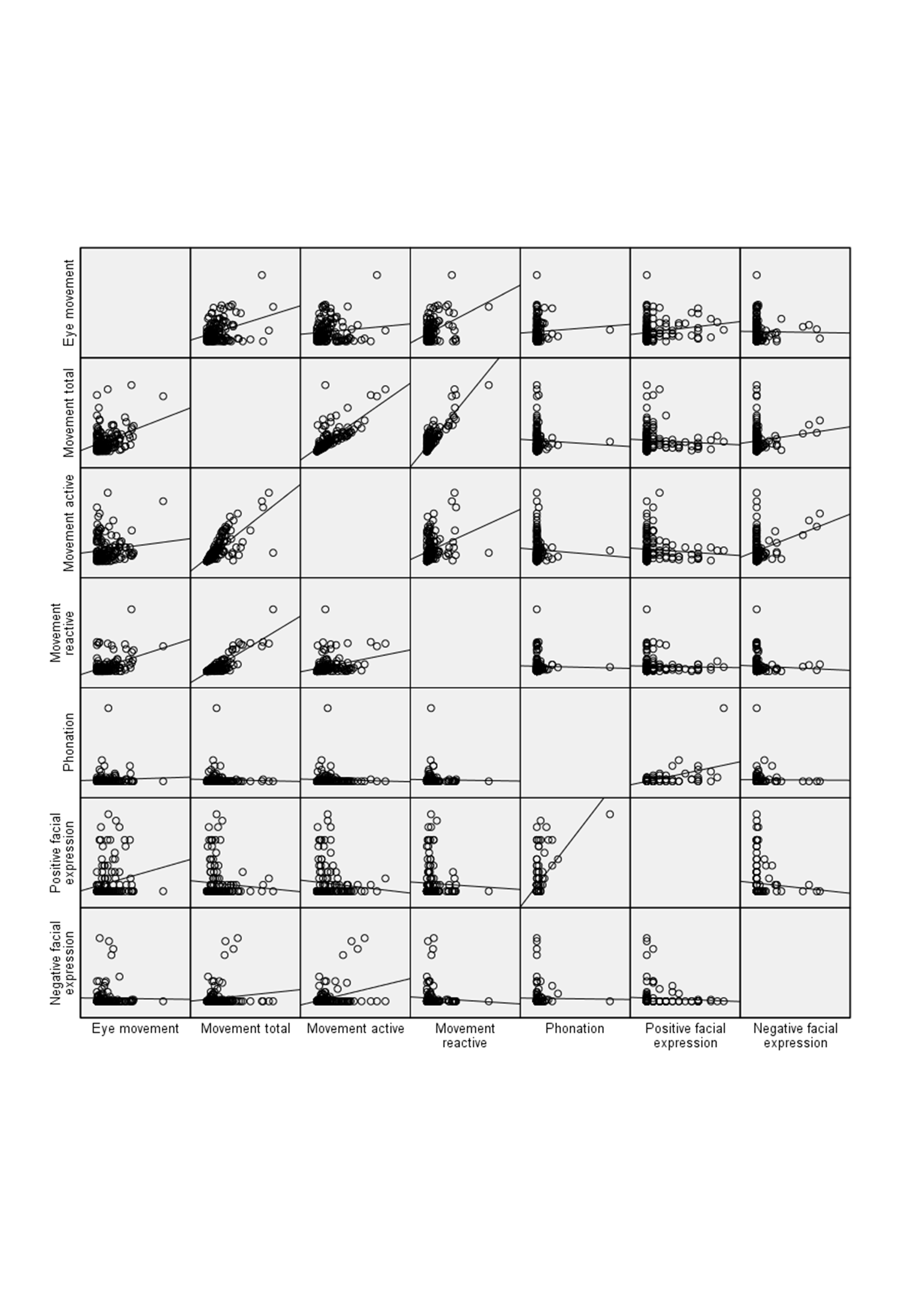

Supplement: S8 Fig — (TIF) [file pone.0222846.s008.tif]
