# Supplementary material for: Animal-assisted therapy for patients in a minimally conscious state: A randomized two treatment multi-period crossover trial
Source: PLoS One. 2019 Oct 1;14(10):e0222846. doi: 10.1371/journal.pone.0222846 (PMC6772068; doi:10.1371/journal.pone.0222846)
Supplement: S1 Table — (DOCX) [file pone.0222846.s009.docx]

**S1 Table. Sessions held by the different therapists.**

| **Therapist ID** | **Total sessions** | **AAT sessions** | **Control sessions** | **AAT sessions (%)** | **Control sessions (%)** |
| --- | --- | --- | --- | --- | --- |
| 7 | 23 | 11 | 12 | 0.48 | 0.52 |
| 2 | 22 | 13 | 9 | 0.59 | 0.41 |
| 17 | 22 | 11 | 11 | 0.50 | 0.50 |
| 16 | 14 | 3 | 11 | 0.21 | 0.79 |
| 10 | 13 | 9 | 4 | 0.69 | 0.31 |
| 9 | 12 | 6 | 6 | 0.50 | 0.50 |
| 8 | 10 | 5 | 5 | 0.50 | 0.50 |
| 15 | 8 | 3 | 5 | 0.38 | 0.63 |
| 3 | 7 | 3 | 4 | 0.43 | 0.57 |
| 4 | 6 | 4 | 2 | 0.67 | 0.33 |
| 14 | 4 | 3 | 1 | 0.75 | 0.25 |
| 1 | 2 | 1 | 1 | 0.50 | 0.50 |
| 11 | 2 | 1 | 1 | 0.50 | 0.50 |
| 13 | 2 | 2 | 0 | 1.00 | 0.00 |
| 18 | 2 | 1 | 1 | 0.50 | 0.50 |
| 19 | 1 | 1 | 0 | 1.00 | 0.00 |
| 20 | 1 | 1 | 0 | 1.00 | 0.00 |

AAT: animal-assisted therapy.
