# Supplementary material for: Animal-assisted therapy for patients in a minimally conscious state: A randomized two treatment multi-period crossover trial
Source: PLoS One. 2019 Oct 1;14(10):e0222846. doi: 10.1371/journal.pone.0222846 (PMC6772068; doi:10.1371/journal.pone.0222846)
Supplement: S2 Table — (DOCX) [file pone.0222846.s010.docx]

**S2 Table. Allocation of therapy sessions to different days of the week.**

| **Weekday** |  | **Total sessions** | **AAT sessions** | **Control sessions** | **AAT sessions (%)** | **Control sessions (%)** |
| --- | --- | --- | --- | --- | --- | --- |
| Monday |  | 30 | 13 | 17 | 0.43 | 0.57 |
| Tuesday |  | 31 | 13 | 18 | 0.42 | 0.58 |
| Wednesday |  | 35 | 22 | 13 | 0.63 | 0.37 |
| Thursday |  | 36 | 15 | 21 | 0.42 | 0.58 |
| Friday |  | 19 | 10 | 9 | 0.53 | 0.47 |

AAT: animal-assisted therapy
