# Supplementary material for: Animal-assisted therapy for patients in a minimally conscious state: A randomized two treatment multi-period crossover trial
Source: PLoS One. 2019 Oct 1;14(10):e0222846. doi: 10.1371/journal.pone.0222846 (PMC6772068; doi:10.1371/journal.pone.0222846)
Supplement: S3 Table — (DOCX) [file pone.0222846.s011.docx]

**S3 Table. Correlations of the analyzed behaviors.**

|  |  | **Eye movement** | **Movement total** | **Movement active** | **Movement reactive** | **Phonation** | **Positive facial expression** | **Negative facial expression** |
| --- | --- | --- | --- | --- | --- | --- | --- | --- |
| **Eye movement** | r | 1 | 0.35* | 0.11 | 0.41 | 0.05 | 0.18 | -0.01 |
|  | p-value |  | < 0.001 | 0.172 | < 0.001* | 0.543 | 0.025* | 0.866 |
| **Movement total** | r | 0.35 | 1 | 0.74 | 0.86 | -0.04 | -0.07 | 0.13 |
|  | p-value | < 0.001* |  | < 0.001* | < 0.001* | 0.650 | 0.384 | 0.125 |
| **Movement active** | r | 0.11 | 0.74 | 1 | 0.30 | -0.02 | -0.09 | 0.31 |
|  | p-value | 0.172 | < 0.001* |  | < 0.001* | 0.819 | 0.276 | < 0.001 |
| **Movement reactive** | r | 0.41 | 0.86 | 0.30 | 1 | -0.02 | -0.03 | -0.05 |
|  | p-value | < 0.001* | < 0.001* | < 0.001* |  | 0.819 | 0.677 | 0.512 |
| **Phonation** | r | 0.05 | -0.04 | -0.05 | -0.02 | 1 | 0.52 | -0.01 |
|  | p-value | 0.543 | 0.650 | 0.582 | 0.819 |  | < 0.001* | 0.915 |
| **Positive facial expression** | r | 0.18 | -0.07 | -0.09 | -0.03 | 0.52 | 1 | -0.06 |
|  | p-value | 0.025* | 0.384 | 0.276 | 0.677 | < 0.001* |  | 0.431 |
| **Negative facial expression** | r | -0.01 | 0.13 | 0.31 | -0.05 | -0.01 | 0.43 | 1 |
|  | p-value | 0.866 | 0.125 | < 0.001* | 0.512 | 0.915 | 0431 |  |

r: Pearson correlation coefficient; *statistically significant
