# Supplementary material for: Animal-assisted therapy for patients in a minimally conscious state: A randomized two treatment multi-period crossover trial
Source: PLoS One. 2019 Oct 1;14(10):e0222846. doi: 10.1371/journal.pone.0222846 (PMC6772068; doi:10.1371/journal.pone.0222846)
Supplement: S2 File — (PDF) [file pone.0222846.s013.pdf]

Clinical Study Protocol

Study title: Evaluating the short-term effects of animal-assisted therapy on the rehabilitation process of patients with severe disorders of consciousness (DOC) at REHAB Basel: a randomised-controlled trial

Short Title: AAT REHAB schwere Bewusstseinsstörung

Study Type:	Clinical trial without Investigational Medicinal Product
Study Categorisation:	A
Study Registration:	Intended registries: "Swiss National Clinical Trials Portal" (SNCTP) and "clinicaltrials.gov"
Study Identifier:	AAT REHAB schwere Bewusstseinsstörung
Sponsor, Sponsor-Investigator or Principal Investigator:	REHAB Basel<br>Im Burgfelderhof 40 / Postfach<br>4012 Basel
Intervention:	Animal-assisted therapy
Protocol Version and Date:	Version 1.0, 25.03. 2015

CONFIDENTIAL

The information contained in this document is confidential and the property of the REHAB Basel. The information may not - in full or in part - be transmitted, reproduced, published, or disclosed to others than the applicable Competent Ethics Committee(s) and Regulatory Authority(ies) without prior written authorisation from the sponsor except to the extent necessary to obtain informed consent from those who will participate in the study.

Signature Page(s)

Study number	Registration number will be provided after registration at SNCTP and clinical-trial.gov
Study Title	Evaluating the short-term effects of animal-assisted therapy on the rehabilitation process of patients with severe disorders of consciousness at REHAB Basel: a randomised-controlled trial

The Sponsor-Investigator has approved the protocol version 1, 25.03.2015, and confirm hereby to conduct the study according to the protocol, current version of the World Medical Association Declaration of Helsinki, ICH-GCP guidelines or ISO 14155 norm if applicable and the local legally applicable requirements.

Sponsor-Investigator:
REHAB Basel
PD Dr. med. Margret Hund-Georgiadis, Chefärztin und medizinische Leiterin, REHAB Basel

Place/Date Signature

Principal Investigator:
Dr. Karin Hediger

--- --

Place/Date Signature

Local Principal Investigator at study site*:

I have read and understood this trial protocol and agree to conduct the trial as set out in this study protocol, the current version of the World Medical Association Declaration of Helsinki, ICH-GCP guidelines or ISO 14155 norm and the local legally applicable requirements.

Site	REHAB Basel, Im Burgfelderhof 40, 4012 Basel
Principal investigator	Dr. Karin Hediger

Place/Date   Signature

**Note:* In multicentre studies, this page must be individually signed by all participating Local Principal Investigators.

Table of Contents

STUDY SYNOPSIS 7
STUDY SUMMARY IN LOCAL LANGUAGE 10
ABBREVIATIONS 11
STUDY SCHEDULE 12
1. STUDY ADMINISTRATIVE STRUCTURE 13
1.1 Sponsor, Sponsor-Investigator 13
1.2 Principal Investigator(s) 13
1.3 Laboratory 13
1.4 Monitoring institution 13
1.5 Data Safety Monitoring Committee 14
1.6 Any other relevant Committee, Person, Organisation, Institution 14
2. ETHICAL AND REGULATORY ASPECTS 15
2.1 Study registration 15
2.2 Categorisation of study 15
2.3 Competent Ethics Committee (CEC) 15
2.4 Competent Authorities (CA) 15
2.5 Ethical Conduct of the Study 15
2.6 Declaration of interest 15
2.7 Patient Information and Informed Consent 15
2.8 Participant privacy and confidentiality 16
2.9 Early termination of the study 16
2.10 Protocol amendments 16
3. BACKGROUND AND RATIONALE 17
3.1 Background and Rationale 17
3.2 Investigational Product (treatment, device) and Indication 17
3.3 Preclinical Evidence 17
3.4 Clinical Evidence to Date 17
3.5 Dose Rationale / Medical Device: Rationale for the intended purpose in study (pre-market MD)17
3.6 Explanation for choice of comparator (or placebo) 17
3.7 Risks / Benefits 17
3.8 Justification of choice of study population 18
4. STUDY OBJECTIVES 18
4.1 Overall Objective 18
4.2 Primary Objective 18
4.3 Secondary Objectives 18
4.4 Safety Objectives 18
5. STUDY OUTCOMES 19
5.1 Primary Outcome 19
5.2 Secondary Outcomes 19
5.3 Other Outcomes of Interest 19
5.4 Safety Outcomes 19
6. STUDY DESIGN 20
6.1 General study design and justification of design 20
Animal-assisted therapy sessions 21
Control sessions 21

6.2 Methods of minimising bias 21
Randomisation 21
Blinding procedures 21
Other methods of minimising bias 22
6.3 Unblinding Procedures (Code break) 22
7. STUDY POPULATION 23
7.1 Eligibility criteria 23
7.2 Recruitment and screening 23
7.3 Assignment to study groups 23
7.4 Criteria for withdrawal / discontinuation of participants 23
8. STUDY INTERVENTION 24
8.1 Identity of Investigational Products (treatment / medical device) 24
Experimental Intervention (treatment / medical device) 24
Control Intervention (standard/routine/comparator treatment / medical device) 24
Packaging, Labelling and Supply (re-supply) 24
Storage Conditions 24
8.2 Administration of experimental and control interventions 24
Experimental Intervention 24
Control Intervention 24
8.3 Dose / Device modifications 24
8.4 Compliance with study intervention 25
8.5 Data Collection and Follow-up for withdrawn participants 25
8.6 Trial specific preventive measures 25
8.7 Concomitant Interventions (treatments) 25
8.8 Study Drug / Medical Device Accountability 25
8.9 Return or Destruction of Study Drug / Medical Device 25
9. STUDY ASSESSMENTS 26
9.1 Study flow chart(s) / table of study procedures and assessments 26
9.2 Assessments of outcomes 27
Assessment of primary outcome 27
Assessment of secondary outcomes 27
Assessment of other outcomes of interest 27
Assessment of safety outcomes 27
Assessments in participants who prematurely stop the study 28
9.3 Procedures at each visit 28
Screening visit, Day -14: informed consent is gathered. Demographics and inclusion/exclusion criteria are derived from the electronic health record. 28
Pre-intervention, Day -5 to -1: questionnaire with the relatives is done 28
Intervention visits 3-18, Day 0 - 21: 28
Follow-up, Day 22 +/-5: questionnaire with the relatives and the therapist is done. 28
10. SAFETY 29
10.1 Category A: Clinical trial without Investigational Medicinal Product 29
Definition and Assessment of safety related events 29
Reporting of Safety related events 29
11. STATISTICAL METHODS 30
11.1 Hypothesis 30

11.2 Determination of Sample Size 30
11.3 Statistical criteria of termination of trial 30
11.4 Planned Analyses 30
Datasets to be analysed, analysis populations 30
Primary Analysis 30
Secondary Analyses 31
Interim analyses 31
Safety analysis 31
Deviation(s) from the original statistical plan 31
11.5 Handling of missing data and drop-outs 31
12. QUALITY ASSURANCE AND CONTROL 32
12.1 Data handling and record keeping / archiving 32
Case Report Forms 32
Specification of source documents 32
Record keeping / archiving 32
12.2 Data management 32
Data Management System 32
Data security, access and back-up 32
Analysis and archiving 32
Electronic and central data validation 33
12.3 Monitoring 33
12.4 Audits and Inspections 33
12.5 Confidentiality, Data Protection 33
12.6 Storage of biological material and related health data 33
13. PUBLICATION AND DISSEMINATION POLICY 33
14. FUNDING AND SUPPORT 34
14.1 Funding 34
14.2 Other Support 34
15. INSURANCE 34
16. REFERENCES 35
17. APPENDICES 36

STUDY SYNOPSIS

**Sponsor / Sponsor-Investigator**	REHAB Basel<br>PD Dr. med Margret Hund-Geogiadis
**Study Title:**	Evaluating the short-term effects of animal-assisted therapy on the rehabilitation process of patients with severe disorders of consciousness at REHAB Basel: a randomised-controlled trial
**Short Title / Study ID:**	AAT REHAB schwere Bewusstseinsstörung
**Protocol Version and Date:**	Version 1, 25.3.2015
**Trial registration:**	Intended registration at "Swiss National Clinical Trials Portal" (SNCTP) und "clinicaltrials.gov".
**Study category and Rationale**	Category A: Clinical trial without Investigational Medicinal Product
**Clinical Phase:**	Not applicable (no clinical trial with drug or medical device study)
**Background and Rationale:**	It is critical to establish effective, early treatment models for patients with disorders of consciousness after intensive care that can facilitate recovery of consciousness and physical function. At REHAB Basel there is the possibility to treat all patients with animal-assisted therapy.<br>Regarding patients with brain-injuries that are in a further rehabilitation phase we do have first promising results that show that these people can profit from animal-assisted therapy.<br>There is practical evidence but no scientific investigations that persons with severe disorders of consciousness can profit from animal-assisted therapy regarding their level of awareness. Therefore, this study investigates this question in a pilot study with randomised-controlled design. The study wants to provide first insights in effects of animal-assisted therapy and to perhaps find new ways to effectively treat patients with severe disorders of consciousness.
**Objective(s):**	The aim of this study is to investigate the effect of animal-assisted therapy on the degree of awareness of inpatients at REHAB Basel with severe disorders of consciousness.
**Outcome(s):**	Brief statement of primary study outcome and the main secondary study outcome measures.<br>Primary Outcome: patient's awareness assessed via behavioral video coding<br>Secondary Outcomes:<br>1) Heart rate and heart rate variability<br>2) BAVESTA Score after the therapy sessions<br>3) Therapist's assessment via questionnaire
**Study design:**	Controlled cross-over study, within-subject trial with repeated measurement

**Inclusion / Exclusion criteria:**	Study population are inpatients at REHAB Basel with severe disorders of consciousness who rehabilitated at REHAB Basel. Severity of the disorder of consciousness is assessed via the Coma Recovery Scale (Koma Remissions Skala, KRS) and the BAVESTA Scale. Patients included in this study are in a minimal conscious state.<br>Exclusion criteria are medical contraindications (allergies, phobias, etc.) or if the patient's medication changes radically during the time of data collection.
**Measurements and procedures:**	The study intervention is animal-assisted therapy. The therapy sessions are standardised sessions using a therapy animal while doing physiotherapy, occupational therapy or speech therapy. Control sessions are comparable therapy interventions without an animal present.<br>Sessions take place four time a week for four weeks (8 intervention sessions and 8 control sessions) and last for about 20 minutes.<br>Measurements are: behaviour assessed via video coding during the therapy session, heart rate and heart rate variability assessed via EKG during the therapy sessions and the BAVESTA Score as well as a questionnaire for the therapists after each therapy session.
**Study Product / Intervention:**	As therapy animals, small and trained animals (guinea pigs and rabbits) will be used.
**Control Intervention (if applicable):**	The control condition is the comparable "standard" therapy session without the presence of an animal: physiotherapy, occupational therapy or speech therapy.
**Number of Participants with Rationale:**	10 participants will be recruited.<br>This number was determined on one side in regard to the pilot character of the study and on the other side on the basis of feasibility regarding working with severe disorders of consciousness. Another reference was the current experiences of a first pre-analysis of the AAT REHAB study with patients with brain injuries (Short-term effects of animal-assisted therapy on the rehabilitation process of brain-injured patients at REHAB Basel). We analysed the first 6 patients and found a significant effect of animal-assisted therapy on positive emotions.
**Study Duration:**	1.5 year
**Study Schedule:**	May 2015 - Nov 2016

**Investigator(s):**	Dr. Karin Hediger, Swiss TPH, Socinstrasse 57, Postfach, 4002 Basel & REHAB Basel, Im Burgfelderhof 40, Postfach, 4012 Basel<br>karin.hediger@unibas.ch / K.Hediger@rehab.ch<br>079 519 78 85<br><br>PD Dr. med. Margret Hund-Georgiadis<br>Chefärztin und medizinische Leiterin<br>REHAB Basel, Im Burgfelderhof 40, Postfach, 4012 Basel<br>M.Hund@rehab.ch<br>+41 (0)61 325 00 00
**Study Centre(s):**	Single-centre<br>REHAB Basel, Im Burgfelderhof 40, Postfach, 4012 Basel
**Statistical Considerations:**	A mixed effect model will be used to calculate the outcomes. Covariates as gender and rehabilitation state will be included. 10 patients will be included in this pilot study.
**GCP Statement:**	This study will be conducted in compliance with the protocol, the current version of the Declaration of Helsinki, the ICH-GCP or ISO EN 14155 (as far as applicable) as well as all national legal and regulatory requirements.

STUDY SUMMARY IN LOCAL LANGUAGE

Ziel der Untersuchung ist es, die Wirkungen von tiergestützter Therapie auf das beobachtbare Verhalten von Menschen mit schweren Bewusstseinsstörungen zu untersuchen. Dazu erhalten 10 Patienten des REHAB Basel, die sich im minimal conscious state befinden, über 4 Wochen lang pro Woche jeweils zwei Therapieeinheiten "herkömmliche" Therapie (dies sind entweder Ergotherapie, Logopädie oder Physiotherapie) sowie zwei Therapieeinheiten "tiergestützte" Therapie. Die Inhalte der jeweils zwei vergleichbaren Therapieeinheiten sind weitgehend vergleichbar, so dass sie sich durch die Anwesenheit eines Tieres unterscheiden.

Erhoben wird einerseits das Ausmass des Bewusstseins der Patienten während der Therapie. Dieses wird über das gezeigte Verhalten definiert. Um dieses zu erfassen, werden alle Therapieeinheiten auf Video aufgenommen und anschliessend mittels Observer kodiert. Weiter wird die Herzrate und die Herzratenvariabilität der Patienten während den Therapiesitzungen gemessen und am Ende jeder Therapiesitzung der BAVETA Score sowie die Einschätzung der Therapeuten in einem Fragebogen erhoben.

Auf diese Weise wird untersucht, ob das Bewusstsein der Patienten, die sich im minimal conscious state befinden, durch die Anwesenheit eines Tieres stärker fördern lässt im Vergleich zu bisherigen Standardtherapien.

ABBREVIATIONS

AAT	Animal-assisted therapy
AE	Adverse Event
BAVESTA	Basler Vegetative State Assessment
CA	Competent Authority (e.g. Swissmedic)
CEC	Competent Ethics Committee
CRF	Case Report Form
ClinO	Ordinance on Clinical Trials in Human Research *(in German: KlinV, in French: OClin)*
DOC	Disorder of consciousness
eCRF	Electronic Case Report Form
CTCAE	Common terminology criteria for adverse events
DSUR	Development safety update report
GCP	Good Clinical Practice
IB	Investigator’s Brochure
Ho	Null hypothesis
H1	Alternative hypothesis
HFG	Humanforschungsgesetz (Law on human research)
HMG	Heilmittelgesetz
HRA	Federal Act on Research involving Human Beings
IMP	Investigational Medicinal Product
IIT	Investigator-initiated Trial
ISO	International Organisation for Standardisation
ITT	Intention to treat
KlinV	Verordnung über klinische Versuche in der Humanforschung *(in English: ClinO, in French OClin)*
KRS	Koma Remissions Skala, Coma Recovery Scale
LPTh	Loi sur les produits thérapeutiques
LRH	Loi fédérale relative à la recherche sur l’être humain
MD	Medical Device
OClin	Ordonnance sur les essais cliniques dans le cadre de la recherche sur l'être humain *(in German : KlinV, in English : ClinO)*
PI	Principal Investigator
SDV	Source Data Verification
SOP	Standard Operating Procedure
SPC	Summary of product characteristics
SUSAR	Suspected Unexpected Serious Adverse Reaction
TMF	Trial Master File

STUDY SCHEDULE

Study Periods	Screening	Pre-Intervention	Intervention Period				Follow-up
Visit	1	2	3-6	7-10	11-14	15-18	19
Time (hour, day, week)	-14d	-1d	0	7d	14d	21d	22+/-5d
Patient Information and Informed Consent	X						
Demographics	X						
In- /Exclusion Criteria	X						
Randomisation			X				
Questionnaire relatives		x					X
Primary Variables			X	x	x	X	
Secondary Variables			X	x	x	x	
Intervention			X	x	x	X	
Adverse Events			X	x	x	x	

1. STUDY ADMINISTRATIVE STRUCTURE

1.1 Sponsor, Sponsor-Investigator

Sponsor-Investigator:

PD Dr. med. Margret Hund-Georgiadis

Chefärztin und medizinische Leiterin

Fachärztin FMH Neurologie

REHAB Basel
Zentrum für Querschnittgelähmte und Hirnverletzte
Im Burgfelderhof 40, Postfach, CH-4012 Basel
Tel. +41 61 325 00 00
Fax +41 61 325 00 01
rehab@rehab.ch

Involved in planning the study, data collection and management, analysis, and interpretation of data as well as writing of the report.

1.2 Principal Investigator(s)

Dr. phil. Karin Hediger

1: REHAB Basel, Im Burgfelderhof 40, Postfach, CH-4012 Basel
Tel. +41 61 325 00 00

Tel. direct: +41 61 325 59 05

2: Human and Animal Health Unit
Department of Epidemiology and Public Health
Swiss Tropical and Public Health Institute
Socinstrasse 57, 4002 Basel, Switzerland
Tel: +41 61 284 87 28
Mobile:+41 79 519 78 85
email: karin.hediger@unibas.ch

Involved in planning the study, data collection and management, analysis, and interpretation of data as well as writing of the report.

Address and telephone number(s) of the trial site:

REHAB Basel, Im Burgfelderhof 40, Postfach, CH-4012 Basel
Tel. +41 61 325 00 00

1.3 Laboratory

Not applicable. There is no laboratory involved.

1.4 Monitoring institution

The monitoring institution is the sponsor: REHAB Basel, Switzerland.

1.5 Data Safety Monitoring Committee

Not applicable. There is no Data Safety Monitoring Commitee involved.

1.6 Any other relevant Committee, Person, Organisation, Institution

Counselling/Collaborators:

Prof. Dr. Jakob Zinsstag, Human and Animal Health Unit, Deputy head, Swiss Tropical and Public Health Instutite, Socinstrasse 57, 4051 Basel

PD Dr. Dennis C. Turner, IEMT Schweiz c/o Swiss TPH, Socinstrasse 57, 4051 Basel

Prof. Dr. Jens Gaab, Psychologisches Institut, Universität Basel

2. ETHICAL AND REGULATORY ASPECTS

The decision of the CEC and Swissmedic/foreign competent authority concerning the conduct of the study will be made in writing to the Sponsor-Investigator before commencement of this study. The clinical study can only begin once approval from all required authorities has been received. Any additional requirements imposed by the authorities shall be implemented.

2.1 Study registration

The study will be registered at "Swiss National Clinical Trials Portal“ (SNCTP) und “clinicaltrials.gov“.

2.2 Categorisation of study

Risk category: A

2.3 Competent Ethics Committee (CEC)

The responsible investigators ensure that approval from "Ethikkommission Nordwest- und Zentralschweiz" (EKNZ) is sought for the clinical study.

No changes are made to the protocol without prior Sponsor and CEC approval, except where necessary to eliminate apparent immediate hazards to study participants.

Premature study end or interruption of the study is reported to EKNZ within 15 days. The regular end of the study is reported to the EKNZ within 90 days, the final study report shall be submitted within one year after study end. Amendments are reported according to chapter 3.10.

2.4 Competent Authorities (CA)

Not applicable.

2.5 Ethical Conduct of the Study

The study will be carried out in accordance to the protocol and with principles enunciated in the current version of the Declaration of Helsinki, the guidelines of Good Clinical Practice (GCP) issued by ICH, in case of medical device: the European Directive on medical devices 93/42/EEC and the ISO Norm 14155 and ISO 14971, the Swiss Law and Swiss regulatory authority’s requirements. The CEC and regulatory authorities will receive annual safety and interim reports and be informed about study stop/end in agreement with local requirements.

Moreover, the investigators as well as REHAB Basel follow the declarations and guidelines of the international Association of Human-Animal-Organisations (IAHAIO), in particular the "Prague declaration" (available at www.iahaio.org).

2.6 Declaration of interest

All involved persons declare no conflict of interest with any financial organization regarding the research of this study.

2.7 Patient Information and Informed Consent

The investigators will explain to each legal representative of the participant the nature of the study, its purpose, the procedures involved, the expected duration, the potential risks and benefits and any discomfort it may entail. Each legal representative of the participant will be informed that the participation in the study is voluntary and that he/she may withdraw from the study at any time and that withdrawal of consent will not affect his/her subsequent medical assistance and treatment.

The legal representative of the participant must be informed that his/her medical records may be examined by authorised individuals other than their treating physician.

All legal representatives of the participants for the study will be provided a participant information sheet and a consent form describing the study and providing sufficient information for participant to make an informed decision about their participation in the study. Each legal representative has at least two days to decide whether to participate or not.

The patient information sheet and the consent form will be submitted to the CEC and to the competent authority (as applicable) to be reviewed and approved. The formal consent of a participant, using the approved consent form, must be obtained before the participant is submitted to any study procedure.

The legal representative of the participant should read and consider the statement before signing and dating the informed consent form, and should be given a copy of the signed document. The consent form must also be signed and dated by the investigator (or his designee) and it will be retained as part of the study records.

2.8 Participant privacy and confidentiality

The investigator affirms and upholds the principle of the participant's right to privacy and that they shall comply with applicable privacy laws. Especially, anonymity of the participants shall be guaranteed when presenting the data at scientific meetings or publishing them in scientific journals.

Individual subject medical information obtained as a result of this study is considered confidential and disclosure to third parties is prohibited. Subject confidentiality will be further ensured by utilising subject identification code numbers to correspond to treatment data in the computer files.

For data verification purposes, authorised representatives of the Sponsor (-Investigator), a competent authority (e.g. Swissmedic), or an ethics committee may require direct access to parts of the medical records relevant to the study, including participants' medical history.

2.9 Early termination of the study

The Sponsor-Investigator may terminate the study prematurely according to certain circumstances, for example:

- ethical concerns,
- insufficient participant recruitment,
- when the safety of the participants is doubtful or at risk, respectively,
- early evidence of benefit or harm of the experimental intervention

Participation in the study of individual participants may be terminated according to the following reasons:

- the legal representative of the patient/the patient wishes to stop participation
- medical problems due to contact to an animal
- ethical concerns
- when the safety of the involved animal is doubtful or at risk

2.10 Protocol amendments

Substantial amendments are only implemented after approval of the EKNZ.

Under emergency circumstances, deviations from the protocol to protect the rights, safety and well-being of human subjects may proceed without prior approval of the sponsor and the EKNZ. Such deviations shall be documented and reported to the sponsor and the EKNZ as soon as possible.

All Non-substantial amendments are communicated to the EKNZ as soon as possible if applicable and to the EKNZ within the Annual Safety Report (ASR).

Only the sponsor or the investigator is allowed to amend the protocol or to provide suggestions for a protocol amendment.

3. BACKGROUND AND RATIONALE

3.1 Background and Rationale

The study aim is to investigate the effects of animal-assisted therapy for patients with severe disorders of consciousness. There is practical evidence but no scientific investigations that including animals in therapy might help to stimulate the patient's awareness. Therefore this study wants to provide first insights in effects of animal-assisted therapy and to perhaps find new ways to effectively treat patients with severe disorders of consciousness.

3.2 Investigational Product (treatment, device) and Indication

The intervention is animal-assisted therapy. Due to internationally defined terms (see www.iahaio.org) this is a specific and goal-oriented therapeutic treatment in the presence of an animal. At REHAB Basel animal-assisted physiotherapy, animal-assisted speech therapy and animal-assisted occupational therapy is used within the rehabilitation process of patients with brain injuries and, more specific, also with patients with severe disorders of consciousness.

3.3 Preclinical Evidence

There are other rehabilitation clinics like the one for children in Zürich that uses animal-assisted therapy for patients with brain-injuries and also for patients with severe disorders of consciousness. Practical experiences are promising.

3.4 Clinical Evidence to Date

Although there is a growing number of hospitals and rehabilitation centres that use animal-assisted therapy for patients with severe disorders of consciousness, there is not a lot of data available.

A case study with dog-assisted therapy concluded that this can be a reasonable option to treat patients with severe brain damages (Bard, Bard, & Kornhuber, 2013).

3.5 Dose Rationale / Medical Device: Rationale for the intended purpose in study (pre-market MD)

There are no guidelines on dosage and treatment periods for AAT. The dosage of twice a week and treatment period of four weeks was chosen with regard to practical experiences at REHAB Basel and after discussion with the involved staff.

3.6 Explanation for choice of comparator (or placebo)

The comparator was chosen as most comparable as possible. Because the purpose of the study is to investigate the effect of the presence of an animal, the same standardized therapy interventions except for that there is no animal present serve as comparator.

3.7 Risks / Benefits

This study leads to a better understanding of the effects of animal-assisted therapy for patients with severe disorders of consciousness. And on this basis, future patients can profit because the study results may lead to developing effective new therapies for this patient group and implementing them in the rehabilitation process in health-care facilities.
With respect to direct benefits, it must be stated that the participants don't profit directly (apart from the expected outcomes) by participating in the study, and there is no compensation. The patients would also have the possibility to participate in the animal-assisted therapy program without taking part in this study.
Because no medical treatments or invasive measurement procedures are applied, we do not expect any risks for the participants that require additional security parameters. The only risks are possible injuries (scratches, bites), allergies or exposure to zoonoses. To minimize that risk, routine procedures such as training, appropriate housing and care as well as regular veterinary medical checks are used, not only for this study, but also within the normal animal-assisted therapeutic program of REHAB Basel. In a recent survey done at REHAB Basel we found that there were no such problems during the last year while working animal-assisted.
In our opinion, the knowledge gained in potential rehabilitation-enhancing effects of animal-assisted therapy for patients with severe disorders of consciousness far exceeds the small risk of animal-

inflicted injury or zoonotic disease. These risks associated with interacting with animals are also accepted by an increasing number of hospitals, nursing homes, psychiatric and rehabilitation clinics, prisons and schools because of the observed benefits.

3.8 Justification of choice of study population

The study population are inpatients of REHAB Basel with severe disorders of consciousness, defined by a minimal conscious state. Therefore there are vulnerable participants.

We decided to include patients with severe disorders of consciousness because there is an urgent need for interventions. This patient population has the least interventions during rehabilitation due to limiting factors of their state. For the rehabilitation process it is necessary that the therapists have more possibilities to get in contact with the patients. Animal-assisted therapy is one of such a new intervention method. We think that it is essential to investigate effects of such a therapy for the further development of already existing therapy concepts or creation of new ones.

In every case there is informed consent obtained from the legal representative to ensure the patient can participate in the study. The legal representative is informed oral and written and has at least two or three days time to give his or her consent.

Every signs and symptoms showing that the participant is unwilling to participate in the study results in the participant being excluded from participation. If the participant can be elucidated later, when he or her is capable of judgment or with the help of accessible relatives the whole study procedure is explained to him or her and he or her is free to recall. This will lead in cancellation of the obtained data.

It is guaranteed that a physician not participating in the study, safeguards participant interest and insures proper medical care.

4. STUDY OBJECTIVES

4.1 Overall Objective

The purpose of this study is to evaluate the effects of animal-assisted therapy on patients with severe disorders of consciousness. The study aims to describe the effects that integrating an animal into a "standard therapy" has in comparison to this standard setting.

4.2 Primary Objective

The study seeks primarly to determine the effect of the presence of an animal and the interaction with an animal of the patient's degree of awareness.

4.3 Secondary Objectives

The secondary objective is to assess if these effect is reflected in a biological correlate (heart rate and heart rate variability) and in a standardized measurement like the BAVESTA.

4.4 Safety Objectives

Because in this study there are no treatments with medicine and no invasive measurement techniques, we do not expect any risks to the participants that require additional security parameters. Moreover, patients are already under constant medical observation and treatment at REHAB Basel which allow the detection and treatment of any complication, even after the conclusion of the study. Additional security assessments are not necessary.

At the beginning of each AAT session the emotional status of the involved animal will be assessed for its fitness to interact with the patient. In the event of aggression from the animal or the human patient, AAT would be interrupted.

Direct adverse effects like allergies or injuries (e.g. scratches or bites) are collected and immediately reported to medical staff.

5. STUDY OUTCOMES

5.1 Primary Outcome

The primary endpoint is the patient's degree of awareness during the therapy session assessed via behavioral video coding.

5.2 Secondary Outcomes

Secondary endpoints will be the heart rate and heart rate variability during the therapy sessions as well as rating's from the therapists regarding the patient's behaviour assessed via the BAVESTA and a questionnaire.

5.3 Other Outcomes of Interest

Before starting the therapy sessions with data collection, the relatives of the patient, will - if possible - be questioned about their expectations and assumptions of animal-assisted therapy in a semi-structured interview. After finishing the 24 sessions, the patient's relatives and the therapist will be interrogated in a semi-structured interview to also assess qualitative data that include subjective perceived positive and negative effects, attributions of effects, the perceived role of animals, etc.

5.4 Safety Outcomes

Because in this study there are no treatments with medicine and no invasive measurement techniques, we do not expect any risks to the participants that require additional security parameters. Moreover, patients are already under constant medical observation and treatment at REHAB Basel which allow the detection and treatment of any complication, even after the conclusion of the study. Additional security assessments are not necessary.

At the beginning of each AAT session the emotional status of the involved animal will be assessed for its fitness to interact with the patient. In the event of aggression from the animal or the human patient, AAT would be interrupted.

Direct adverse effects are collected and immediately reported to medical staff. This can be:

- allergies
- fear
- bites
- scratches

6. STUDY DESIGN

6.1 General study design and justification of design

This study is designed as a controlled cross-over, within-subject trial with repeated measurement. The experimental condition is the standardised therapy session using therapy animals (AAT), while the control condition is the comparable "standard" therapy session without the presence of an animal.

Over a period of four weeks, 10 patients with severe disorders of consciousness have four standardised therapy sessions per week, that alternate in a way that two sessions in two consecutive weeks are similar except for one is with the presence of animals and one without (see table 1). In this way, data is collected over 16 therapy sessions (8 experimental, 8 control) for each patient.

Each therapy session lasts 15 minutes with an additional 15 minutes after the session for filling in the BAVESTA and the questionnaire (see table 3).

Table 1: Therapy session process (colour and numbers stand for comparable sessions)

	Monday	Tuesday	Wednesday	Thursday	Friday
Week1	Ergo + AAT 1	Ergo 2	Logo + AAT 3	Logo 4	
Week2	Ergo 1	Ergo + AAT 2	Logo 3	Logo + AAT 4	
Week3	Ergo + AAT 5	Ergo 6	Logo + AAT 7	Logo 8	
Week4	Ergo 5	Ergo + AAT 6	Logo 7	Logo + AAT 8	

The study will take place at REHAB Basel. Animal-assisted therapies will be held at a special room prepared for the presence of animals.

Patients will be selected and allocated randomly to start with either the experimental or the control condition.

Although it is unclear to what extent patients understand words, they cannot be fully blinded because they see if the animal is present or not. But the patients do not know what hypotheses we have, for example, that animal-assisted therapy increases social behaviour, and what the exact outcome measures are. Investigators cannot be blinded because the presence of the animal is clearly visible.

Table 2: Course of events

	What	Who
	Decision based on medical point of view on which patients might participate	By the head of department at REHAB
	Screening, selecting and randomising patients	By the head of department at REHAB together with the investigators
	Patient information and informed consent	
Data collection	Semi-structured interview	With relatives (if consenting)
	18 therapy sessions (over 4 weeks)	
	Semi-structured interview	With relatives (if consenting) and the therapist

Table 3: Assessments during a session

What	When	Who
Videotaping	During the whole therapy session	Study team
Heart rate and heart rate variability	During the whole therapy session	Study team
BAVESTA	At the end of the therapy session	Therapist
Questionnaire	At the end of the therapy session	Therapist

Animal-assisted therapy sessions

The animals involved are guinea pigs and rabbits that are housed and looked for in the "Therapie-Tiergarten" at REHAB Basel. All animals were specially selected for their use in animal-assisted therapy and trained by the handlers. They are all accustomed to patients with severe disorders of consciousness. During the whole therapy sessions, all animals have the possibility to retreat from the activities. Security of patients as well as of animals must be ensured at any time. Responsibility for patients lies with the therapists while the handler has the responsibility for the animals.

The arrangement and contents of the therapy sessions are selected by the therapists with regard to the patient-specific therapeutic aims. But as physical contact is an important factor for building a relationship, the therapist should ensure that every patient is able to touch the animal directly during each therapy session.

The therapist was well as the handler will be present during the whole therapy session for safety reasons and to give the patient and the animal a feeling of security. The handler stands in the background as long as his interventions are not needed.

Control sessions

Each animal-assisted therapy session has a corresponding control therapy session, with the same therapist, which is comparable with respect to content and setting as far as possible. The therapist decides on the content and format of the session so that a mostly high level of comparability is given.

Limitations of the design

Since the study is designed as within-subject trial, it is only possible to look at short-term effects.

6.2 Methods of minimising bias

Randomisation

In collaboration with REHAB Basel, potential patients are selected by a screening and then allocated randomly to their first session with data collection either in the presence of an animal or not. The numbers for randomisation are generated in excel producing random numbers. Because of the within-subject design we only have to randomize for the starting condition.

Blinding procedures

Although it is unclear to what extent patients understand words, they cannot be fully blinded because they see if the animal is present or not. But the patients do not know what hypotheses we have, for example, that animal-assisted therapy increases social behaviour, and what the exact outcome measures are.

Students that code the videos cannot be blinded because the presence of the animal is clearly visible. Data analysis of physical parameters however can be done blind.

Other methods of minimising bias

To assess the short-term effects during an animal-assisted therapy session, each patient serves as his own control.

The videotaped therapy sessions are coded by independent observers that are trained in the coding procedure. Because observations cannot be made fully blind there is a very stiff coding protocol and an inter-rater reliability with a kappa higher than .8 is ensured.

All therapy sessions of one patient will take place at the same time of the day to minimize diurnal influences.

6.3 Unblinding Procedures (Code break)

Because there is no blinding process, no unblinding procedures need to be described

7. STUDY POPULATION

Describe in the subchapters below the population to be studied; this should include a description of the study settings if relevant (e.g., out-patients, community clinic, academic hospital) and list of centres/countries where data will be collected (or reference to where list of study sites can be obtained). Provide plan of actions to be taken if the enrolment goals are not met.

7.1 Eligibility criteria

Participants fulfilling all of the following inclusion criteria are eligible for the study, for example:

- Informed Consent as documented by signature (Appendix Informed Consent Form)
- Inpatients of REHAB Basel
- Disorder of consciousness: minimal conscious state defined via:
- KRS Score of auditory 3-4 or visual 2-5 or motor 3-5 or oromotor/verbal = 3 or communication = 1
- BAVESTA Score of 2.8

BAVESTA Score:2.8 / KRS Scores:

The presence of any one of the following exclusion criteria will lead to exclusion of the participant, for example:

- Enrolment of the investigator, his/her family members, employees and other dependent persons,
- medical contraindications for contact with animals as allergy, phobia etc.
- If the patient's medication changes radically during the time of data collection

7.2 Recruitment and screening

Patients are recruited at REHAB Basel. Possible patients are identified by involved therapists or physicians. They are then screened for exclusion criterias. When a patient is chosen as eligible, the therapist that works most with the patient explains the study to the patient and his relatives to obtain informed consent.

The study participants do not receive payment or compensation.

7.3 Assignment to study groups

All participants receive both the intervention and the control treatment. Randomization concerns the starting condition.

7.4 Criteria for withdrawal / discontinuation of participants

Participation in the study will be cancelled if a patient or his legal representative wishes to do so or if a patient chooses to withdrawal from the animal-assisted therapy program. Other criteria for withdrawal are if the patient is harmed by an animal of if the animals are abused by the patient.

In case of withdrawal of study patients, more patients will be recruited until the planned number of patients is reached.

8. STUDY INTERVENTION

8.1 Identity of Investigational Products (treatment / medical device)

Experimental Intervention (treatment / medical device)

The experimental intervention is speech therapy, physiotherapy and/or occupational therapy in the presence of an animal, the so called animal-assisted therapy. As animals, we use guinea pigs and rabbits. All animals were specially selected for their use in animal-assisted therapy and trained by the handlers. They are all accustomed to brain-injured patients. During the whole therapy sessions, all animals have the possibility to retreat from the activities. Security of patients as well as of animals must be ensured at any time. Responsibility for patients lies with the therapists while the handler has the responsibility for the animals.

The arrangement and contents of the therapy sessions are selected by the therapists with regard to the patient-specific therapeutic aims. But as physical contact is an important factor for building a relationship, the therapist should ensure that every patient is able to touch the animal directly during each therapy session.

The therapist was well as the handler will be present during the whole therapy session for safety reasons and to give the patient and the animal a feeling of security. The handler stands in the background as long as his interventions are not needed.

Control Intervention (standard/routine/comparator treatment / medical device)

Each animal-assisted therapy session has a corresponding control therapy session (speech therapy, occupational therapy and/or physiotherapy), with the same therapist, which is comparable with respect to content and setting as far as possible. The therapist decides on the content and format of the session so that a mostly high level of comparability is given.

Packaging, Labelling and Supply (re-supply)

Not applicable.

Storage Conditions

Not applicable.

8.2 Administration of experimental and control interventions

Experimental Intervention

The experimental intervention takes place twice a week for four weeks (see overview time course of events). Each session lasts for 20 minutes.

Control Intervention

The experimental intervention takes place twice a week for four weeks (see overview time course of events). Each session lasts for 20 minutes.

8.3 Dose / Device modifications

Not applicable.

8.4 Compliance with study intervention

Each therapy session is videotaped and the compliance is therefore evident.

8.5 Data Collection and Follow-up for withdrawn participants

There is no follow-up for withdrawn patients.

8.6 Trial specific preventive measures

There are no treatment restrictions.

8.7 Concomitant Interventions (treatments)

There are no treatment restrictions.

8.8 Study Drug / Medical Device Accountability

Not applicable.

8.9 Return or Destruction of Study Drug / Medical Device

Not applicable.

9. STUDY ASSESSMENTS

9.1 Study flow chart(s) / table of study procedures and assessments

Study Periods	Screening	Pre-Intervention	Intervention Period				Follow-up
Visit	1	2	3-6	7-10	11-14	15-18	19
Time (hour, day, week)	-14d	Between -5 and -1d	0	7d	14d	21d	22+/-5d
Patient Information and Informed Consent	X						
Demographics	X						
In- /Exclusion Criteria	X						
Randomisation			x				
Questionnaire relatives		x					X
Primary Variables			x	x	X	X	
Secondary Variables			x	x	X	X	
Intervention			x	x	X	X	
Adverse Events			x	x	X	X	

Table 1: Intervention period (days are exemplary and can change in reality)

	**Monday**	**Tuesday**	**Wednesday**	**Thursday**	**Friday**
Week1	Session 1	Session 2	Session 3	Session 4	
Week2	Session 5	Session 6	Session 7	Session 8	
Week3	Session 9	Session 10	Session 11	Session 12	
Week4	Session 13	Session 14	Session 15	Session 16	

Primary Outcome:

During the intervention period, all 16 sessions are videotaped in whole and afterwards analysed via behaviour coding in Noldus Observer.

Secondary Outcomes:

During the Intervention period, heart rate and heart rate variability is measured during the 16 therapy sessions. After each therapy session, the therapists assess the patient's behaviour during the session via the BAVESTA and in a structured questionnaire.

Other Outcomes

The semistructured questionnaire for the relatives is done before starting the intervention period. After the intervention period, a semistructured questionnaire is done again with the relatives and also the therapists.

9.2 Assessments of outcomes

Assessment of primary outcome

Primary outcome is the degree of the patient's self or environment awareness during the therapy session. This is assessed via videotaping the whole therapy session. After data collection, the patientt's behavior is coded using Noldus Observer System and a predefined coding scheme.

Assessment of secondary outcomes

Heart rate and heart rate variability: measured as beats per minute during the whole therapy session, assessed via EKG by using the Polar System.

BAVESTA: Basler Vegetative State Assessment

Therapist's rating of the patient's behaviour: via a structured questionnaire.

Assessment of other outcomes of interest

The semistructured questionnaire for the relatives is done before starting the intervention period. After the intervention period, a semistructured questionnaire is done again with the relatives and also the therapists.

Assessment of safety outcomes

At the beginning of each AAT session the emotional status of the involved animal will be assessed for its fitness to interact with the patient. In the event of aggression from the animal or the human patient, AAT would be interrupted.

Direct adverse effects like allergies or injuries (e.g. scratches or bites) are collected and immediately reported to medical staff.

9.2.1.1 Adverse events

Direct adverse effects like allergies or injuries (e.g. scratches or bites) are collected and immediately reported to medical staff.

9.2.1.2 Laboratory parameters

Not applicable. Every patient is under constant medical surveillance anyway.

9.2.1.3 Vital signs

Not applicable. Every patient is under constant medical surveillance anyway.

Assessments in participants who prematurely stop the study

There are no follow-up assessments.

9.3 Procedures at each visit

Study Periods	Screening	Pre-Intervention	Intervention Period				Follow-up
Visit	1	2	3-6	7-10	11-14	15-18	19
Time (hour, day, week)	-14d	Between -5 and -1d	0	7d	14d	21d	22+/-5d
Patient Information and Informed Consent	X						
Demographics	X						
In- /Exclusion Criteria	X						
Randomisation			x				
Questionnaire relatives		x					X
Primary Variables			x	x	X	X	
Secondary Variables			x	x	X	x	
Intervention			x	x	X	X	
Adverse Events			x	x	X	x	

Screening visit, Day -14: informed consent is gathered. Demographics and inclusion/exclusion criteria are derived from the electronic health record.

Pre-intervention, Day -5 to -1: questionnaire with the relatives is done

Intervention visits 3-18, Day 0 - 21:

At each visit the following procedure is done:

Heart rate is assessed via Polar, the session is videotaped and at the end of the session, the therapists fills in the questionnaire.

Follow-up, Day 22 +/-5: questionnaire with the relatives and the therapist is done.

10. SAFETY

10.1 Category A: Clinical trial without Investigational Medicinal Product

Definition and Assessment of safety related events

Animal-assisted therapy is a non-invasive and established therapy. The only risks are possible injuries (scratches, bites), allergies or exposure to zoonoses. To minimize that risk, routine procedures such as training, appropriate housing and care as well as regular veterinary medical checks are used, not only for this study, but also within the normal animal-assisted therapeutic program of REHAB Basel. In a recent survey done at REHAB Basel we found that there were no such problems during the last year while working animal-assisted.

Reporting of Safety related events

Reporting to Sponsor-Investigator:

Health hazard that require measures are reported to the Sponsor-Investigator within 24 hours upon becoming aware of the event:

Reporting to Authorities:

Health hazard that require measures are reported to the EKNZ within 2 days upon becoming aware of the event:

11. STATISTICAL METHODS

11.1 Hypothesis

Null Hypothesis:

The perceptive functions of patients with severe disorders of consciousness do not differ in sessions with an animal present in comparison to sessions without an animal present.

Alternative Hypothesis:

The perceptive functions of patients with severe disorders of consciousness are higher in sessions with an animal present in comparison to sessions without an animal present.

11.2 Determination of Sample Size

10 patients will be included in the trial.

This number was determined on one side in regard to the pilot character of the study and on the other side on the basis of feasibility regarding working with patients with severe disorders of consciousness as well as the current experiences of a first pre-analysis of the AAT REHAB study with patients with brain injuries (Short-term effects of animal-assisted therapy on the rehabilitation process of brain-injured patients at REHAB Basel). We analysed the first 6 patients and found a significant effect of animal-assisted therapy on positive emotions.

11.3 Statistical criteria of termination of trial

The trial will be terminated after 10 patients have received 8 sessions of animal-assisted therapy and 8 sessions of "standard"-therapy.

11.4 Planned Analyses

To account for the correlated data structure within patients and over time, data will be analysed using generalised mixed models. The individual patient will be defined as random effect. The outcomes are expected to be approximately normally distributed and will be defined as fixed effect.

Datasets to be analysed, analysis populations

Data from all participants who completed the 16 sessions will be included in the final analyses, data will be analysed using available case approach. No data will be imputed for missing values, i.e. individuals lost to follow up.

Descriptice analyses will be conducted. For categorical variables, descriptive analyses will consist of raw numbers and percentages, for continuous variables the mean, median, standard deviation, and range will be reported.

Primary Analysis

The primary outcome is the patient's perceptive functions that is assessed via behaviour coding. Data are percentages. With these data, generalised mixed models are run.

Relevant covariates that might influence the main outcome measure are age, gender, and the achieved gain in patient's functioning (assessed via the FIM and BAVESTA score gain before and after the intervention)

For all analyses, the niveau of significance will be 5% (p </= 0.05). Results with a probability of error equal to or lower than 10% (p </= 0.10) will be indicated as trends.

Analyses are done by the principal investigator and master's students at the end of the trial.

Secondary Analyses

The secondary outcomes are calculated in the same way as the primary outcome.

Interim analyses

There are interim analyses that are done for the students master's thesis. There are several master students working in the study and they have the right to analyse data at the time when they need to finish their master's thesis with respect to their scientific question.

There are no stopping guidelines with respect to interim analyses.

Safety analysis

Not applicable

Deviation(s) from the original statistical plan

Deviations from the planned analyses can only be made by the Sponsor-Investigator or the principal investigator. Deviations will be reported to the EKNZ in the annual report.

11.5 Handling of missing data and drop-outs

Data will be analysed using an available case approach. No data will be imputed for missing values, i.e. for individuals lost to follow up.

12. QUALITY ASSURANCE AND CONTROL

Quality assurance and quality control is maintained via double-checking by different involved study personnel (investigator and students).

The principal Investigator is responsible for proper training of all involved study personnel. This is secured via written instructions and procedures that every student has access to.

There are regularly meetings where procedures are discussed together and new information is shared. There is also a communication system for new information to share.

12.1 Data handling and record keeping / archiving

Case Report Forms

Case report forms are electronic Excel sheets. Electronic data is inserted from the programs directly into these excel files and paper data is transferred onto the Excelfiles. Participant numbers are used for identification. Authorized to enter data are only the investigator and the involved students. Each participant does have a identifying form with biographic data such as birth date and name of relatives. This sheet is stored together with the form of the informed consent.

Specification of source documents

Source data are available at the site to document the existence of the study participants. They include electronic health records at REHAB Basel (Sytems: OPALE, KIS) as well as paper health records, both of them not study specific. The study specific source data are informed consent forms and documentation of each observed session. These are found in the office of the study team at REHAB Basel.

Record keeping / archiving

All study data are archived for 10 years after study termination or premature termination of the clinical trial. Data will be stored at REHAB Basel secured from unauthorised persons and will be destroyed after the statutory period of record keeping.

12.2 Data management

Data entry is made by students working in the projects. All data that are on paper are stored originally and transferred into an excel file. All data that is gathered electronically is stored in the specific study folder. Data entry is controlled by another student.

All data are transferred on a regular basis onto a second electronic storage. Files are numbered and labelled with date.

Data Management System

There is no Data Management System used.

Data security, access and back-up

Access to the data have only members of the study team: investigator and involved students.

Back-up systems: two separate hard disks

Analysis and archiving

Data analysis is done by the students and the investigator as well as by the involved statician.

Electronic and central data validation

Data are validated using filtering systems in Excel, by double-checking and via descriptive statistical analyses.

12.3 Monitoring

There is no special study-specific monitoring planned.

The source data/documents are accessible to monitors and questions are answered during monitoring.

12.4 Audits and Inspections

There are no special study-specific audits and inspections planned.

The study documentation and the source data/documents are accessible to auditors/inspectors (also CEC and CA) and questions are answered during inspections. All involved parties must keep the participant data strictly confidential.

12.5 Confidentiality, Data Protection

The obtained data will be handled strictly confidentially. Access to the protocol have only the investigators and the study team (all students sign a contract of confidentiality). Personal data will be made anonymous for data analysis. No names will be published at any time, and published reports will not allow for identification of single subjects. Confidentiality and anonymity will be ensured throughout.

Direct access to source documents will be permitted for purposes of monitoring (12.3), audits and inspections (12.4) (ICHE6, 6.10).

12.6 Storage of biological material and related health data

Not applicable.

13. PUBLICATION AND DISSEMINATION POLICY

The trial results are communicated inhouse at REHAB Basel in the Wissenschaftsforum and via a short Publication in the REHAB Info Newsletter. Patients who wish to be informed, can leave their new address and will receive a summary of the results.

Results will also be presented at international congresses (field of rehabilitation, psychology and animal-assisted interventions) and published in an international journal. If possible, an open-access publication is desirable.

14. FUNDING AND SUPPORT

14.1 Funding

This study has financial support from REHAB Basel and from a personal grant to Dr. Karin Hediger from the Forschungsfonds Basel.

14.2 Other Support

There is no other support.

15. INSURANCE

Insurance will be provided by the Sponsor. A copy of the certificate is filed in each investigator site file and the trial master file.
Animal-assisted therapy is an integral component of the therapeutic concept at REHAB Basel. Potential risks and liability claims related to animal-assisted therapy are therefore covered by the REHAB Basel.

16. REFERENCES

1. Bard, S., Bard, M., & Kornhuber, M. E. (2013). Dog-Based Multi-Sensorial Therapy of a Patient with a "Persistent Vegetative State" - A Case Report. *Rehabilitation, 52*, 399-405.
2. Seel, R. T., Douglas, J., Dennison, A. C., Heaner, S., Farris, K. and Rogers, C. (2013) Specialized early treatment for persons with disorders of consciousness: program components and outcomes. *Arch Phys Med Rehabil,* 94, 1908-1923.
3. Declaration of Helsinki, Version October 2013, (http://www.wma.net/en/30publications/10policies/b3/index.html)
4. International Conference on Harmonization (ICH, 1996) E6 Guideline for Good Clinical Practice. (http://www.ich.org/fileadmin/Public_Web_Site/ICH_Products/Guidelines/Efficacy/E6_R1/Step4/E6_R1__Guideline.pdf)
5. International Conference on Harmonization (ICH, 1997) E8 Guideline: General Considerations for Clinical Trials http://www.ich.org/fileadmin/Public_Web_Site/ICH_Products/Guidelines/Efficacy/E8/Step4/E8_Guideline.pdf)
6. Humanforschungsgesetz, HFG Bundesgesetz über die Forschung am Menschen (Bundesgesetz über die Forschung am Menschen, HFG) vom 30. September 2011/ Loi fédérale relative à la recherche sur l'être humain (loi relative à la recherche sur l'être humain, LRH) du 30 septembre 2011. (http://www.bag.admin.ch/themen/medizin/00701/00702/07558/index.html?lang=de)
7. Verordnung über klinische Versuche in der Humanforschung (Verordnung über klinische Versuche, KlinV) vom 20. September 2013 / Ordonnance sur les essais cliniques dans le cadre de la recherche sur l'être humain (Ordonnance sur les essais cliniques, OClin) du 20 septembre 2013. (http://www.bag.admin.ch/themen/medizin/00701/00702/12310/index.html?lang=de)
8. Heilmittelgesetz, HMG Bundesgesetz über Arzneimittel und Medizinprodukte (Heilmittelgesetz, HMG) vom 15. Dezember 2000/Loi fédérale sur les médicaments et les dispositifs médicaux (Loi sur les produits thérapeutiques, LPT) du 15 décembre 2000. (http://www.admin.ch/ch/d/sr/8/812.21.de.pdf)
9. ISO 14155:2011 Clinical investigation of medical devices for human subjects -- Good clinical practice (www.iso.org)
10. ISO 10993 Biological evaluation of medical devices (www.iso.org)
11. WHO, International Clinical Trials Registry Platform (ICTRP) (http://www.who.int/ictrp/en/)

17. APPENDICES

1. Case Report Form (CRF)
2. Patient Information and informed consent
3. Questionnaires
